# Continuous Introduction of H5 High Pathogenicity Avian Influenza Viruses in Hokkaido, Japan: Characterization of Viruses Isolated in Winter 2022–2023 and Early Winter 2023–2024

**DOI:** 10.1155/2024/1199876

**Published:** 2024-03-14

**Authors:** Lim Yik Hew, Norikazu Isoda, Fumihito Takaya, Kohei Ogasawara, Daiki Kobayashi, Loc Tan Huynh, Tatsuru Morita, Rio Harada, Nikolay Gennadievich Zinyakov, Dmitriy Borisovich Andreychuk, Ilya Alexandrovich Chvala, Viktor Nikolaevich Irza, Yukiko Watanabe, Hiroko Fujita, Keisuke Saito, Takahiro Hiono, Yoshihiro Sakoda

**Affiliations:** ^1^Laboratory of Microbiology, Department of Disease Control, Faculty of Veterinary Medicine, Hokkaido University, Kita 18, Nishi 9, Kita-Ku, Sapporo 060-0818, Hokkaido, Japan; ^2^International Collaboration Unit, International Institute for Zoonosis Control, Hokkaido University, Kita 20, Nishi 10, Kita-Ku, Sapporo 001-0020, Hokkaido, Japan; ^3^Hokkaido University Institute for Vaccine Research and Development (HU-IVReD), Hokkaido University, Sapporo, Japan; ^4^One Health Research Center, Hokkaido University, Sapporo 060-0818, Hokkaido, Japan; ^5^Botanic Garden, Field Science Center for Northern Biosphere (FSC), Hokkaido University, Sapporo 060-0003, Hokkaido, Japan; ^6^Institute for Raptor Biomedicine Japan Co., Ltd., Kushiro 084-0922, Hokkaido, Japan; ^7^Faculty of Veterinary Medicine, College of Agriculture, Can Tho University, Can Tho 900000, Vietnam; ^8^Federal Center for Animal Health (FGBI ARRIAH), Vladimir 600901, Russia; ^9^Research Faculty of Agriculture, Hokkaido University, Sapporo 060-8589, Hokkaido, Japan

## Abstract

High pathogenicity avian influenza (HPAI) has impacted poultry and wild birds globally. The number of H5 HPAI virus (HPAIV) infection cases in wild birds in Hokkaido (Northern Japan) was high in the last two seasons, contributing to virus spillover to resident birds and poultry. Therefore, H5 HPAIVs in birds and mammals in Hokkaido in winter 2022–2023 and 2023–2024 were monitored and viruses were phylogenetically, antigenically, and pathogenetically characterized. Thirty HPAIV isolates were subtyped and pathotyped by sequencing the hemagglutinin (HA) gene of viruses. Phylogenetic analysis of the HA gene revealed that all isolated HPAIVs were categorized into clade 2.3.4.4b and divided into three groups (G2b, G2c, and G2d). Most isolates belonging to subgroup G2d clustered with isolates in winter 2021–2022 in Hokkaido. The other isolates were categorized into two subgroups, G2b and G2c, mainly composed of isolates in Honshu Island in winter 2021–2022 and 2022–2023, respectively. Two H5 HPAIVs isolated in Eastern Russia in spring and autumn 2022 were genetically close to most Hokkaido isolates (G2d), and a virus isolated in Hokkaido in November 2023 was also grouped in subgroup G2d. Further analysis of all eight gene segments identified six types of gene constellations. Cross-hemagglutination inhibition test indicated that the antigenicity of H5 HPAIVs isolated in the last several seasons was similar within them but slightly different from that in the 2010s. Three chicken breeds were intranasally challenged with four representative isolates to assess their pathogenicity. All chickens except one broiler chicken were dead until 5-day postchallenge with different pathogenicity of these viruses. The pathogenicity of one HPAIV strain was significantly lower in broiler chickens than in layer chickens. The mixture of multiple characteristics of HPAIVs in Hokkaido was confirmed by bird migration routes. Thus, many HPAIVs can be brought and scattered anywhere on Earth.

## 1. Introduction

Avian influenza is a highly contagious disease in poultry caused by influenza type A virus infection [1]. Wild birds, especially migratory ducks, are the natural reservoir for avian influenza virus (AIV). AIVs belonging to genus *Alphainfluenzavirus* and family *Orthomyxoviridae* comprise eight single-stranded, negative-sense, segmented RNAs that can be categorized into 16 and 9 subtypes based on the antigenic characteristics of hemagglutinin (HA) and neuraminidase (NA) surface proteins, respectively [2, 3]. AIVs can naturally spread among wild aquatic birds and infect various bird species, resulting in varying clinical symptoms ranging from low to severe, sometimes leading to increased mortality. AIVs can classified into high pathogenicity (HPAIVs) or low pathogenicity (LPAIVs) according to pathogenicity in chickens [1]. HPAIVs are only restricted to H5 and H7 subtypes because of multiple basic amino acid residues in the cleavage site of the HA protein in natural settings, which can be cleaved by the ubiquitous protease in birds and lead to systemic infection [4, 5].

The worldwide circulation of H5 HPAIVs has contributed to a significant global economic loss due to outbreaks in the poultry industry across Asia, Europe, Africa, and, most recently, North and South America [6–9]. Most H5 HPAIVs are descendants of an isolate A/goose/Guangdong/1/1996 (H5N1) (Gs/Gd) lineage that has been circulating in wild birds for >25 years and has branched into numerous clades and subclades due to selective genetic mutations and reassortment with multiple influenza subtypes [10]. Among those clades, H5 HPAIVs in clade 2.3.4.4 have rapidly spread and constantly caused outbreaks worldwide since 2014 [11]. In particular, H5 HPAIVs in clade 2.3.4.4b have been consistently isolated in Europe and Asia since 2016 [12]. In late spring 2021, a new epidemiologic trend of H5N1 HPAIV was confirmed in Europe by reassortant viruses between clade 2.3.4.4b H5N8 HPAIVs and LPAIVs locally circulated and led to a large number of infections in wild birds and poultry in many European countries [13]. It was further introduced into the Far East, such as Japan, the Republic of Korea, Russia, and China, possibly by bird migrations [14–16].

In Japan, in winter 2021–2022, 25 high pathogenicity avian influenza (HPAI) outbreaks in poultry and 107 cases in wild birds were reported [17]. Four HPAI outbreaks in poultry, 70 cases in wild birds, and two cases in wild mammals from an Ezo red fox (*Vulpes vulpes schrencki*) and a raccoon dog (*Nyctereutes procyonoides*) were confirmed in Hokkaido Prefecture, located in the northernmost part of Japan and facing the Far East parts of Russia which usually have the nesting site of migratory birds [15, 18]. Based on the genetic analysis of HPAIVs isolated in Hokkaido in winter 2021–2022, most H5N1 HPAIVs were genetically close to H5 HPAIVs circulating in Europe and North America in the same winter, demonstrating that the wider dispersion of HPAIVs to both edges of the Eurasian continent in the same season could be originated from the lakes in Siberia, where migratory birds nest in summer [15].

In summer 2022, no H5 HPAIV cases were reported in Japan, differing from the situation in Europe, where H5 HPAIVs were sustained throughout summer 2022. However, HPAIVs were reintroduced back to Japan at the beginning of the new winter season 2022–2023. Subsequently, 242 HPAI cases and 84 outbreaks were reported in wild birds and poultry, respectively [19], implying that Japan should still be at high risk of HPAIV introduction. The continual introduction of H5N1 HPAIVs with various genetic features may display a different level of pathogenicity in chickens, with slight antigenic differences even within clade 2.3.4.4b H5 HPAIVs [9]. Thus, in this study, H5 HPAIVs isolated from wild birds, mammals such as foxes, and poultry in Hokkaido in winter 2022–2023 were genetically analyzed to obtain epidemiologic findings relating to HPAIV incursion to Hokkaido. Furthermore, the antigenic difference of isolates was analyzed to determine whether the antigenicity of H5 HPAIVs had changed during maintenance in birds, and the pathogenicity of four representative HPAIV strains was experimentally assessed in chickens.

## 2. Materials and Methods

### 2.1. Fecal and Swab Sample Collection, Virus Isolation, and Identification

Two hundred fourteen fecal samples of wild waterfowls were collected during the active survey at Lake Komuke (latitude: 44°25′47′′N; longitude: 143°50′25′′E) and Notsuke Peninsula (latitude: 43°36′12′′N; longitude: 145°17′35′′E) in Eastern Hokkaido in October 2022. The collected fecal samples were mixed with a virus transport medium consisting of minimum essential medium (Nissui, Tokyo, Japan) containing 10 mg/mL streptomycin (Meiji Seika Pharma, Tokyo, Japan), 10,000 U/mL penicillin G (Meiji Seika Pharma, Tokyo, Japan), 250 U/mL nystatin (Sigma–Aldrich, St. Louis, MO, USA), 0.3 mg/mL gentamicin (MSD, Tokyo, Japan), and 0.5% bovine serum albumin fraction V (Roche, Basel, Switzerland) and inoculated into 10-day-old embryonated eggs for virus isolation [20]. To identify the host species, DNA was extracted from the fecal sample using NucleoSpin® DNA Stool (Takara bio, Shiga, Japan) and 749 base pairs near the 5′-terminus of the cytochrome *c* oxidase (COI) gene of a host bird were amplified using the primers described previously [21]. The polymerase chain reaction (PCR) product was sequenced and compared to the sequence data of the COI gene obtained from Barcode of Life Data Systems (http://www.barcodinglife.com). For the passive survey of HPAIV infections in dead animals in Hokkaido, tracheal swabs collected from dead birds, organ homogenates from dead foxes, and lung homogenates of dead chickens in poultry farms with HPAI outbreaks were inoculated into 10-day-old embryonated eggs for virus isolation. Lung samples from the deceased wild fox (Ezo red fox) were homogenized using a Multi-Beads Shocker (Yasui Kikai, Osaka, Japan) to prepare 10% suspension in a virus transport medium. Lung homogenates of dead chickens from HPAI-affected poultry farms were kindly provided by the Ishikari livestock hygiene services in Hokkaido.

Virus propagation in the collected allantoic fluid was confirmed by the hemagglutination assay. The isolated viruses were subtyped by an hemagglutination inhibition (HI) test using chicken hyperimmune sera against the referenced influenza virus strain for subtyping [3]. Two H5N1 HPAIVs, A/crow/Khabarovsk/776-56/2022 (H5N1) and A/goose/Magadan/2272-5/2022 (H5N1), were isolated from crow in April 2022 and wild goose in October 2022, respectively, in Eastern Russia. To determine the pathogenicity of isolates, viral RNA was extracted from the allantoic fluid using TRIzol LS Reagent (Thermo Fisher Scientific, Waltham, MA, USA) or QIAamp viral RNA mini kit (Qiagen, Hilden, Germany) and sequenced by direct sequencing after PCR using a region-specific primer set to confirm multiple basic amino acid residues at the HA gene, the molecular marker of HPAIV [22]. The full length of the viral genome was amplified by reverse transcription-PCR using gene-specific primers to each of the eight viral gene segments for Sanger sequencing [23]. Each gene was directly sequenced using BigDye Terminator v3.1, Cycle Sequencing Kit (Thermo Fisher Scientific, Waltham, MA, USA), and 3500 Genetic Analyzer (Thermo Fisher Scientific). For next-generation sequencing (NGS) of isolates in Japan, the primer sets described by Ip et al. [24] were used to amplify the whole genome of AIV for NGS analysis together with three primer sets that specifically improve the yield of PB2, PB1, and PA segments (Table S1). Oxford Nanopore libraries were prepared using the NEB Ultra II End Repair/dA-Tailing Module (New England Biolabs, Ipswich, MA, USA) and sequenced on Flongle using the Nanopore Direct cDNA sequencing kit or Ligation Sequencing kit V14 (Oxford Nanopore Technologies, Oxford, England). The obtained reads were mapped and assembled using FluGAS version 2 (World Fusion, Tokyo, Japan). For whole-genome sequencing of isolates in Russia, cDNA synthesis was performed using the cDNA Synthesis Kit (Roche, Basel, Switzerland). The libraries of genome-wide sequencing were prepared by using the commercial Nextera XT Library Prep Kit (Illumina, Hayward, CA, USA) and Nextera XT Index Kits (Illumina, Hayward, CA, USA) and sequenced on MiSeq genetic analyzer (Illumina, Hayward, CA, USA) following the manufacturer's instructions for the resequencing of small genomes. The obtained reads were mapped in GS Reference Mapper v2.7 and de novo assembled in GS De Novo Assembler v2.7.

### 2.2. Genetic Analysis

All whole genomes of 30 HPAIVs isolated in Hokkaido and two HPAIVs from Russia were used in the genetic analysis (Table 1). The nucleotide sequences of the 32 isolates were phylogenetically analyzed based on the maximum likelihood method using the best-fit general time-reversible model of the nucleotide substitution with gamma-distribution rate variation among sites (with four rate categories, *Γ*) according to the Tamura and Nei model [25]. Bootstrap analysis under 1,000 replications was applied to construct the phylogenetic tree in MEGA 7 with default parameters [26]. Sequence data of the gene were compared to the reference nucleotide sequence of the representative H5Nx (with different NA subtypes) in clade 2.3.4.4 strains downloaded from the Global Initiative on Sharing All Influenza Data (GISAID) and GenBank. Basic Local Alignment Search Tool (BLAST) in the GISAID database was used to identify the most homologous nucleotide sequences of HPAIVs isolated in this study.

### 2.3. Antigenic Analysis

The antigenicity of HPAIVs isolated in winter 2022–2023 and an isolate in early winter 2023–2024 was compared to each other and to that in the past season in Japan using a cross-HI test (Table S3). Three representative HPAIV strains isolated in winter 2022–2023 A/chicken/Hokkaido/HU-E001/2022 (Ck/Hok/E001/22; H5N1) isolated from a dead chicken of an HPAI outbreak in a poultry farm in Hokkaido in October 2022, A/large-billed crow/Hokkaido/B003/2022 (Cr/Hok/B003/22; H5N2) isolated from a dead crow in an urban garden in the capital of Hokkaido Prefecture, and A/Eurasian wigeon/Hokkaido/Q71/2022 (Ew/Hok/Q71/22; H5N1) isolated from a fecal sample of wild waterfowl were selected according to the potential antigenic difference based on phylogenetic analysis. Hyperimmune serum against each of these three strains was prepared, as described by Kida and Yanagawa [3]. For antiserum production, 500 *µ*g antigen was mixed with Freund's complete or incomplete adjuvant at the first and second injections, respectively, and injected intramuscularly into the thigh muscle of a naïve chicken twice at 14-day intervals. Fourteen days after the second injection, whole blood was collected from the chickens to obtain the serum. Other antigens in the representative strains in clade 2.3.4.4, such as A/white-tailed eagle/Hokkaido/22-RU-WTE-2/2022 (WTE/Hok/R22/22; H5N1), A/northern pintail/Hokkaido/M13/2020 (H5N8) [27], A/Ezo red fox/Hokkaido/2/2023 (H5N1), A/large-billed crow/Hokkaido/B049/2023 (H5N1), A/chicken/Hokkaido/HU-B102/2023 (Ck/Hok/B102/23; H5N1), A/large-billed crow/Hokkaido/B067/2023 (H5N1), A/chicken/Kumamoto/1-7/2014 (Ck/Kum/1-7/14; H5N8) [28], A/black swan/Akita/1/2016 (Bs/Aki/1/16; H5N6) [29], and A/Muscovy duck/DR Congo/KAF1/2017 (Mdk/DRC/KAF1/17; H5N8) [30], were analyzed between and within clade 2.3.4.4 using antiserum; WTE/Hok/R22/22 (H5N1), Ck/Kum/1-7/14 (H5N8), Bs/Aki/1/16 (H5N6), and Mdk/DRC/KAF1/17 (H5N8) for cross-reactivity of hyperimmune antisera and their corresponding antigens. The Japanese stockpile vaccine strain for H5 HPAIV infection in Japan, A/duck/Hokkaido/Vac-1/2004 (Dk/Hok/Vac-1/04; H5N1), was also used for the cross-HI test [31]. Antigenic cartography was generated based on the HI test estimated using web-based software [32]. The *x–*y coordinates of each antiserum and antigen were obtained by loading the data of the titers of the cross-HI test.

### 2.4. Animal Experiments

Three chicken breeds free from the antibodies against H5 AIV were prepared to assess the pathogenicity of H5 HPAIVs. White Leghorn chickens were hatched from embryonated eggs and fed at Hokkaido University. Thirty-one-week-old Rhode Island Red chickens were kindly provided by the Field Science Center for Northern Biosphere, Hokkaido University (Hokkaido, Japan). Four-week-old Chunky chickens were kindly provided by Nippon White Farm Co., Ltd., (Hokkaido, Japan) and Prifoods Co., Ltd. (Aomori, Japan). Each of the four viruses, Ck/Hok/E001/22 (H5N1), Cr/Hok/B003/22 (H5N2), Ew/Hok/Q71/22 (H5N1), and Ck/Hok/B102/23 (H5N1), was challenged to four White Leghorn or Chunky chickens. Only three viruses, Ck/Hok/E001/22 (H5N1), Cr/Hok/B003/22 (H5N2), and Ew/Hok/Q71/22 (H5N1), were challenged to three Rhode Island Red chickens. All chickens were intranasally challenged with representative HPAIVs at 10^6.0^ times of 50% egg infectious dose (EID_50_) and monitored daily for clinical manifestation for 14 days postchallenge (dpc). Tracheal and cloacal swabs were collected from each bird daily until 7 dpc or its death, and the swabs were suspended in a 2 mL of virus transport medium. The infectious titers of the swabs were expressed in 50% tissue culture infectious dose (TCID_50_), which was determined using Madin–Darby canine kidney cells by observing the cytopathic effect (CPE) of virus-infected cells. All chicken experiments were conducted at the Animal BSL-3 Facility, Faculty of Veterinary Medicine, Hokkaido University, which has been accredited by the Association for Assessment and Accreditation of Laboratory Animal Care International since 2007 and were approved by the Institutional Animal Care and Use Committee of the Faculty of Veterinary Medicine, Hokkaido University with the approval numbers 18-0037, 23-0050, and 23-0053.

### 2.5. Mutational Analysis

Mutational analysis was conducted using ClustalW in GENETYX version 16 (GENETYX Co., Tokyo, Japan) for sequence alignment of Hokkaido isolates for single nucleotide polymorphisms analysis and referring to other previously published mutation of concerns [33].

## 3. Results

### 3.1. Genetic Analysis

In winter 2022–2023, nine of 214 fecal samples of wild waterfowls, 56 swab samples of migratory or resident birds, 15 and 2 lung homogenate samples of dead chickens and wild foxes, respectively, in Hokkaido, were diagnosed as positive for H5 AIV infections by detection of H5 HA of AIV. No other HA subtypes of AIV were isolated from any other samples. All H5 viruses were subtyped as H5N1, except for one H5N2 strain isolated from a dead crow in the capital city of Hokkaido (Cr/Hok/B003/22; H5N2). The pathogenicity of all isolates in chickens was deduced as highly pathogenic by confirming the multiple basic amino acid motif at the HA1/HA2 proteolytic cleavage site on the HA gene. Twenty-nine selected isolates from 82 H5 HPAIVs were composed of six from poultry samples, two from fecal samples of migratory birds, two from fox carcasses, and the remaining 19 isolates from resident birds selected for genetic analysis. Two additional H5N1 HPAIVs isolated in Eastern Russia in spring and autumn 2022 were included for genetic analysis. During the early winter season 2023–2024, an HPAIV was additionally isolated from a resident crow in Hokkaido in November; the virus was also included in the genetic analysis. The whole sequences of all eight gene segments of these 32 HPAIVs were registered in the GISAID database (Table 1). In the COI gene database, the sequence of mitochondrial DNA extracted from fecal samples of two migratory birds showed 100% homology to the COI gene of *Mareca penelope* (Eurasian wigeon).

**Figure 1:**
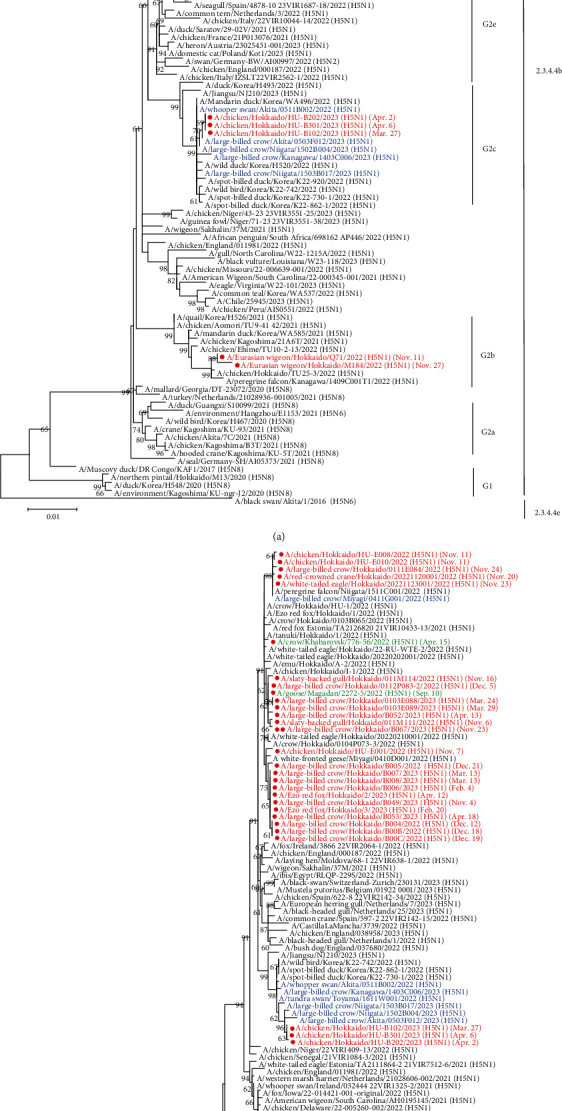
Phylogenetic tree analysis of H5 high pathogenicity avian influenza viruses isolated in the Far East in winter 2022–2023 and early winter 2023–2024. The hemagglutinin (a) and neuraminidase (b) of 29 H5 HPAIVs and an H5 HPAIV isolated in Hokkaido in winter 2022–2023 and early winter 2023–2024, respectively, together with the reference strains in clade 2.3.4.4b and subclades were analyzed using the maximum-likelihood method with MEGA 7. The scale bar represents the number of nucleotide substitutions per site. The number of nodes indicates the probability of confidence levels according to bootstrap analysis based on 1,000 replicates >60%. H5 HPAIVs isolated in Hokkaido in 2022–2023 and early winter 2023–2024 are shown in red, H5 HPAIVs isolated in Eastern Russia in 2022 are in green, and H5 HPAIVs isolated in Honshu Island in 2022–2023 are in blue. The date following the strain's name indicated the collection date of the HPAIVs isolated in this study. The HPAIVs isolated in this study were marked with a circle, and an isolate in early winter 2023–2024 was marked with double circles.

In the phylogenetic analysis of the H5 HA gene, all 32 isolates were categorized in clade 2.3.4.4b and classified into the Group 2 (G2) genotype, which is closely related to clade 2.3.4.4b H5N8 HPAIVs that shared the common ancestor with viruses detected in Europe in late 2020 [34] (Figure 1(a)). Based on the structure of the phylogenetic tree, the G2 genotype was further classified into multiple subgroups (G2a–e). Most isolates in 2022–2023 were entered into the same subgroup, which was tentatively designated as G2d, which contains HPAIVs isolated in Hokkaido in winter 2021–2022 and remarkably also clustered with A/crow/Khabarovsk/776-56/2022 (H5N1) and A/goose/Magadan/2272-5/2022 (H5N1) isolated in Eastern Russia in spring and autumn 2022, respectively. Interestingly, the isolate in Hokkaido in November 2023 was also grouped into subgroup of G2d. Ew/Hok/Q71/22 (H5N1) and A/Eurasian wigeon/Hokkaido/M184/2022 (Ew/Hok/M184/22; H5N1) isolated from fecal samples of wild waterfowls were categorized into a different subgroup of G2 clade (G2b), which was clustered with HPAIVs isolated in southern Japan in winter 2021–2022 [35]. Interestingly, one HPAIV A/chicken/Hokkaido/TU25-3/2022 (Ck/Hok/TU25-3/22; H5N1), which was isolated in the HPAI outbreak in a poultry farm in Hokkaido in May 2022, was also included in the G2b subgroup. At the end of March 2023, three H5 HPAI outbreaks were confirmed in poultry farms in Hokkaido. The causal agents of these outbreaks, Ck/Hok/B102/23 (H5N1), A/chicken/Hokkaido/HU-B202/2023 (H5N1), and A/chicken/Hokkaido/HU-B301/2023 (H5N1), were clustered into another new subgroup, which was tentatively designated as G2c. In this G2c subgroup, Hokkaido isolates were closely clustered with isolates predominantly prevalent in Southern Japan in 2022–2023.

**Figure 2:**
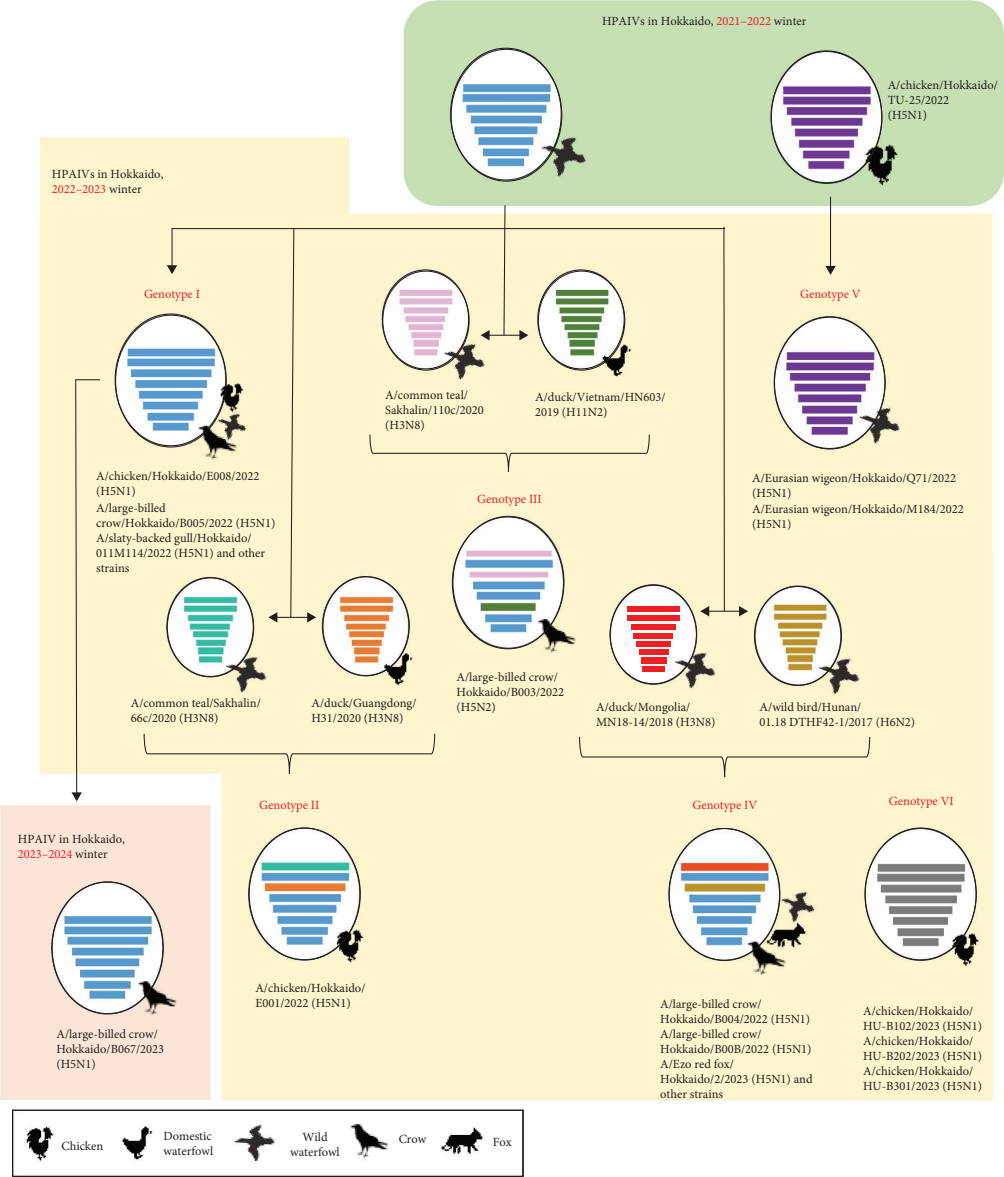
Genome constellation of clade 2.3.4.4b H5 high pathogenicity avian influenza viruses in Hokkaido in winter 2022–2023 and early winter 2023–2024. Oval lines represent avian influenza virus; horizontal bars indicate the eight gene segments (from top to bottom): polymerase basic protein 2, polymerase basic protein 1, polymerase acidic protein, hemagglutinin, nucleoprotein, neuraminidase, matrix protein, and nonstructural protein. Each color represents a different viral origin.

Based on the nucleotide sequence of the HA gene, HPAIVs isolated in Hokkaido were divided into three groups. Similar trends were also confirmed in phylogenetic trees of the N1 NA gene, except for one virus with H5N2 subtype, and other internal genes, such as polymerase basic protein 1 (PB1), nucleoprotein (NP), matrix (M), and nonstructural (NS) gene segments (Figure S1). However, there were different trends in the phylogenetic trees of polymerase basic protein 2 (PB2) and polymerase acidic (PA) genes. In phylogenetic trees of PB2 and PA genes, some isolates, such as Ck/Hok/E001/22 (H5N1), Cr/Hok/B003/22 (H5N2), and A/large-billed crow/Hokkaido/B004/2022 (H5N1), deviated from the major group of Hokkaido isolates in G2d subgroup, indicating that these isolates have undergone reassortment and acquired other wild bird lineages of PB2 and PA genes of LPAIVs (Table S2). Based on BLAST analysis, PB2 and PA genes were highly homologous to the corresponding gene segments of LPAIVs isolated in Far East Asia, including China, Mongolia, and Russia (Table S2). Phylogenetic analysis in all eight gene segments has identified six types of gene constellations of H5 HPAIVs in Hokkaido in 2022–2023 (Figure 2). The subgroup of G2d includes four different gene constellations; a major genotype among isolates in Hokkaido in 2022–2023 (genotype I) has the same gene constellation as the dominant genotype in 2021–2022 in Hokkaido and an isolate in the early winter 2023–2024 in Hokkaido, and two genotypes of two different gene constellations in PB2 and PA gene (genotypes II and IV) and one genotype of three different gene constellations in PB2, PA, and NA (genotype III) were identified as descendants of the dominant genotype in 2021–2022. Ew/Hok/Q71/22 (H5N1) and Ew/Hok/M184/22 (H5N1) in the subgroup of G2b formulated single gene constellation and were designated as genotype V, which also includes Ck/Hok/TU25-3/22 (H5N1). Meanwhile, all the H5 HPAIV isolates in subgroup G2c in Hokkaido also formed a unique gene constellation designated genotype VI.

### 3.2. Antigenic Analysis

**Figure 3:**
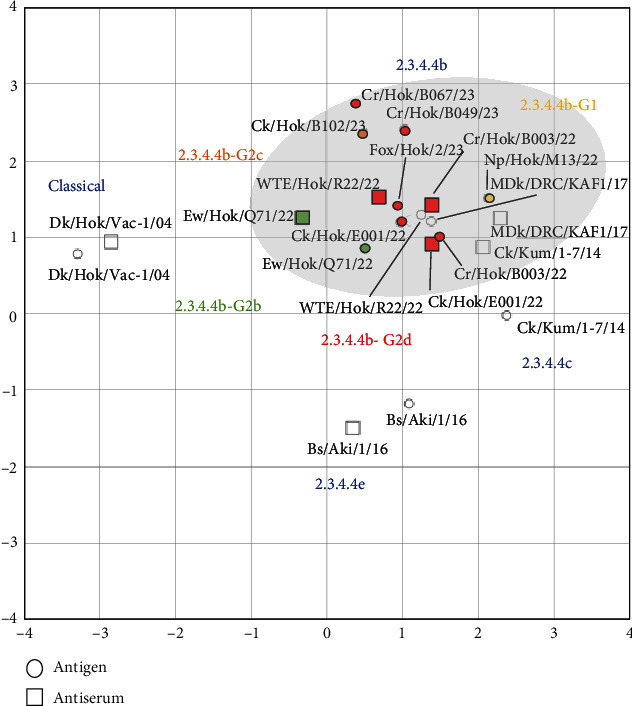
Antigenic map based on cross-hemagglutination inhibition test on clade 2.3.4.4 H5 viruses and the sera. The antigenic relationship among clade 2.3.4.4 H5 viruses, including six viruses in hokkaido in winter 2022–2023 and a virus in early winter 2023–2024 in a different subclade of 2.3.4.4b, was visualized using antigenic cartography based on cross-HI test results, as indicated in Table S3. In the antigenic map, horizontal and vertical axes indicate the antigenic distance of spacing between the grid lines, representing one antigenic unit corresponding to a twofold dilution in the HI assay. Antigen and antiserum in the subgroup of G2d in red, the subgroup of G1 in yellow, the subgroup of G2c in orange, and the subgroup of G2b in green. The highlighted gray area indicates H5 viruses and the sera in clade 2.3.4.4b.

Three viruses, Ck/Hok/E001/22 (H5N1) in subgroup G2d, Cr/Hok/B003/22 (H5N2) in subgroup G2d, and Ew/Hok/Q71/22 (H5N1) in subgroup G2b, were selected for antigenic analysis using the cross-HI test. The antiserum against Ck/Hok/E001/22 (H5N1) showed homologous reaction (same HI titers) with Cr/Hok/B003/22 (H5N2) and Ew/Hok/Q71/22 (H5N1) antigens (Table S3). Meanwhile, the antiserum against Cr/Hok/B003/22 (H5N2) was closely reacted (twofold HI titer differences) with Ck/Hok/E001/22 (H5N1) antigens and Ew/Hok/Q71/22 (H5N1) antigens. The same applies to the antiserum against Ew/Hok/Q71/22 (H5N1), which also displayed close reactivity to the Ck/Hok/E001/22 (H5N1) (twofold HI titer differences) and Cr/Hok/B003/22 (H5N2) (fourfold HI titer differences). Antigenic cartography demonstrated that antigenicity among groups of HPAIVs isolated in winter 2022–2023 and an isolate in November 2023 was almost identical and similar to those of the previously isolated clade 2.3.4.4b (Figure 3). The antigenicity of clade 2.3.4.4b viruses was slightly different from Ck/Kum/1-7/14 (H5N8) in clade 2.3.4.4c. Viruses in clade 2.3.4.4b exhibited more than two antigenic units distant from the vaccine strain: Dk/Hok/Vac-1/04 (H5N1) in the classical clade and Bs/Aki/1/16 (H5N6) in clade 2.3.4.4e.

### 3.3. Pathogenicity Analysis of Four Representative HPAIV Strains in Chickens

**Figure 4:**
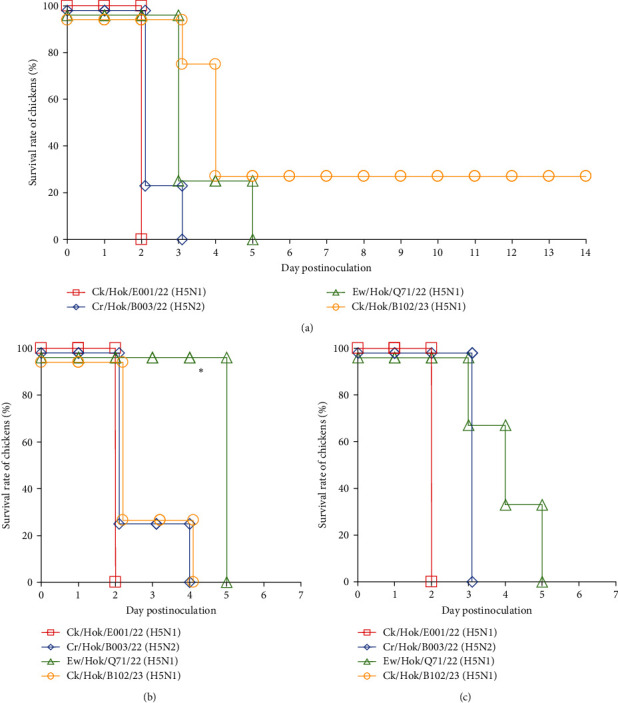
Survival curve of three chicken breeds inoculated with four H5 high pathogenicity avian influenza viruses. Three groups of four 4-week-old Chunky (a) and White Leghorn (b) and three 31-week-old of Rhode Island red (c) chickens were intranasally inoculated with 10^6.0^ EID50/0.1 mL Ck/Hok/E001/22 (H5N1), Cr/Hok/B003/22 (H5N2), Ew/Hok/Q71/22 (H5N1), and Ck/Hok/B102/23, except for Rhode Island Red chickens only challenged with Ck/Hok/E001/22 (H5N1), Cr/Hok/B003/22 (H5N2), and Ew/Hok/Q71/22 (H5N1), respectively, and monitored for 14 days after virus challenge. Clinical manifestation of each chicken was observed, and their survival was recorded daily to calculate the survival rate of each group per day. The survival curve in this figure shows only 14 days of postinoculation for chunky chickens and 7 days of postinoculation for White Leghorn and Rhode Island Red chickens, and the *P*-values for survival rate were calculated using the Kaplan–Meier survival curve log-rank test ( ^*∗*^*P*  < 0.05).

All 4-week-old White Leghorn and Chunky chickens were intranasally challenged with 10^6.0^ EID_50_ of each of the four viruses, and all 31-week-old Rhode Island Red chickens were intranasally challenged with same titer of three viruses. All chickens challenged with Ck/Hok/E001/22 (H5N1) died at 2 dpc, whereas chickens challenged with Cr/Hok/B003/22 (H5N2) died between 2 and 4 dpc (Figure 4). Chickens challenged with Ew/Hok/Q71/22 (H5N1) died between 3 and 5 dpc. Meanwhile, chickens challenged with Ck/Hok/B102/23 (H5N1) started to die between 2 and 4 dpc. Remarkably, one of the chickens inoculated with Ck/Hok/B102/23 (H5N1) survived until 14 dpc, but with no detectable antiserum collected on the last day of the experiment against the virus (Figure 4). Furthermore, no viruses were recovered from the swab samples of the survived chicken throughout 7 days after challenge.

Virus titers in tracheal swabs collected from chickens challenged with Ck/Hok/E001/22 (H5N1) rapidly peaked at 1–2 dpc (Tables 2 and 3 and Table S4). In contrast, virus titers in tracheal swabs of chickens challenged with Cr/Hok/B003/22 (H5N2) at 1 dpc were low (<2.5 log_10_TCID_50_/mL) but rapidly increased at 2–3 dpc (>4.0 log_10_TCID_50_/mL) (Tables 2 and 3 and Table S4). Virus recovery was not confirmed from tracheal swabs of chickens challenged with Ew/Hok/Q71/22 (H5N1) on 1 dpc but confirmed with a steady increase in virus titer from 2 to 5 dpc and the virus titers recovered at the peak of infection was much lower (<4.0 log_10_TCID_50_/mL) when compared to ones after challenge of Ck/Hok/E001/22 (H5N1) and Cr/Hok/B003/22 (H5N2) (Tables 2 and 3 and Table S4). For the virus recovery from the tracheal swabs of chicken challenged with Ck/Hok/B102/23 (H5N1), virus growth was not recognized at 1 dpc (<0.5 log_10_TCID_50_/mL) but rapidly peaked at 2 and 3 dpc (>3.5 log_10_TCID_50_/mL). Interestingly, the virus recovery from the tracheal swabs of chunky chickens challenged with Ck/Hok/B102/23 (H5N1) showed a steady increase in the virus titers from 2 to 4 dpc (Tables 2 and 3). Virus recoveries from cloacal swabs also demonstrated a similar viral load observed in tracheal swabs in all three chicken breeds. The pathogenicity and viral titers observed in swab samples were varied in challenged strains. Although there was variation in the viral loads observed in tracheal and cloacal swabs among the three chicken breeds based on the challenged strains, the growth ability of each challenged virus exhibited among the three chicken breeds remained constant regardless of species and age.

### 3.4. Mutational Analysis

Detailed mutational analysis was performed with 30 isolates in Hokkaido with whole-genome sequences to identify amino acid substitutions that could contribute to virulence, transmissibility, or host adaptation. Thirty-eight variable sites were identified and specifically linked to phenotypes, such as virulence, transmission, or host adaptation, as reported previously [33]. Of these 38 variable sites, eight were identified in PB2, three in PB1, one in PB1-F2 [36], five in PA, eight in HA based on H5 numbering, three in NP, three in M1, and seven in NS1 (Table S5). Due to the introduction of multiple HPAIV genotypes in Hokkaido in 2022–2023, different variables have been found across some genotypes, such as L292V in PB2, S224P in PA, and N319K in NP, previously reported to have contributed to enhancing polymerase activity and virulence in mice [37–39]. Some mutations, such as K207R in PB1 and Q400P in PA, known to decrease polymerase activity in mice, have also been identified in some genotypes [40, 41]. The essential results of the mutation analysis, which possess amino acid variation among the 30 isolates, are summarized in Table 4. Interestingly, one mutation, such as M105V in NP, was exclusively found in all isolates, except for Ew/Hok/Q71/22 (H5N1) and Ew/Hok/M184/22 (H5N1), as this mutation is known to increase pathogenicity in chickens [42]. In addition, the signature of mammalian adaptation gene markers, such as lysine and asparagine at positions 627 and 701 in PB2, was not found in all isolates investigated and deceased Ezo red foxes.

## 4. Discussion

The global spread of clade 2.3.4.4b H5 HPAIVs has caused a large-scale outbreak, significantly impacting the poultry industry and wildlife [32]. The Far East, such as Japan and other countries, has been recognized as a hotspot due to the continuous introduction of HPAIVs. In winter 2021–2022, high numbers of dead wild birds and outbreaks in the poultry farm were affected in Hokkaido due to the introduction of 2.3.4.4b H5N1 HPAIVs [15]. The continuous circulation of H5 HPAIVs in 2023 by migratory birds has disseminated viruses into rarely affected countries in the South American region, such as Peru, Ecuador, and Venezuela [6]. Hokkaido was no exception to the continual invasion of clade 2.3.4.4b H5 HPAIVs in winter 2022–2023. In this study, 30 HPAIVs isolated in Hokkaido were genetically analyzed in all eight gene segments. Based on the phylogenetic analysis of the HA gene segment, all Hokkaido isolates still belonged to clade 2.3.4.4b and further formed three phylogenetic clusters of G2b, G2c, and G2d (Figure 1). HPAIVs in the G2d subgroup share common ancestors closely related to Hokkaido isolates in winter 2021–2022, together with A/crow/Khabarovsk/776-56/2022 (H5N1) and A/goose/Magadan/2272-5/2022 (H5N1) isolated in Eastern Russia in spring and autumn 2022. This might indicate that Hokkaido isolates in winter 2021–2022 in the G2d subgroup had been carried over outside of Hokkaido, including Eastern Russia, by spring migration and could have been maintained in the nested lake in northern Russia during summer 2022. An isolate in Hokkaido in November 2023 was also grouped into the subgroup G2d. The continuous introduction of HPAIVs in the subgroup G2d during the three consecutive seasons could reveal that the viruses in this subgroup should be sustained in the Russian Far East and carried back and forth from Hokkaido, even with three different subgroups (G2b, G2c, and G2d) of H5 HPAIVs been introduced into Hokkaido in winter 2022–2023. This finding can also be supported by the results of gene constellation analysis that genotype I, which is a dominant genotype in Hokkaido in winter 2022–2023, had dominantly caused outbreaks for a certain period [15], was also a majority in winter 2021–2022 and isolated at the beginning of winter 2023–2024. A similar assumption was also proposed for HPAIV isolates in the G2b subgroup. Based on BLAST analysis, some Hokkaido isolates in winter 2022–2023, such as Ew/Hok/Q71/22 (H5N1) and Ew/Hok/M184/22 (H5N1) in the G2b subgroup, showed high sequence identity to Ck/Hok/TU25-3/22 (H5N1) isolated in the poultry outbreak at the end of spring 2022 in Hokkaido. The nucleotide sequences of those isolates were highly homologous to H5 HPAIVs isolated in Southern Japan in winter 2021–2022. These observations also suggested that HPAIV isolates in the G2b subgroup were likely to be brought to Hokkaido by bird migration in spring 2022 and maintained in Northern and Eastern Russia during summer 2022. These HPAIVs were reintroduced into the Far East in the following autumn migration [15]. Multiple HPAI outbreaks caused by H5 HPAIVs in Hokkaido poultry were also reported in early April 2023. One of the isolates from the poultry outbreak, Ck/Hok/B102/23 (H5N1), clustered in G2c subgroup, showed high genetic similarity to isolates from Southern Japan in winter 2022–2023. Those isolates were also genetically close to those circulating in the Republic of Korea and China [16], and invasion of HPAIVs in Southern Japan to Hokkaido was speculated once again in winter 2022–2023 following the spring migration.

During the winter 2022–2023, multiple genotypes of HPAIVs could have been sporadically introduced into Hokkaido. Extensive reassortment events in 2.3.4.4b H5N1 HPAIVs have been frequently reported, such as the recent cases in the Republic of Korea and China, where genetic reassortment was observed between clade 2.3.4.4b H5 HPAIVs with Eurasian LPAIVs [14, 16, 34]. Based on phylogenetic trees of other internal gene segments in this study, it was confirmed that multiple genotypes, genotypes II and III, were reassorted (Figure 2). Reassorted HPAIVs were produced between clade 2.3.4.4b H5 HPAIVs, genotype I, circulating predominantly in Hokkaido in winter 2021–2022, and LPAIVs originated from wild birds in the Far East. It was speculated that dominant H5N1 HPAIVs in European or Asian countries could undergo genetic reassortment with other LPAIVs during migration or at the breeding sites of migratory birds. Thus, further genetic analysis should provide the role of wild birds in contributing to the reshuffle of genetic properties of AIVs, which allows the formation of a temporary genome constellation of viruses that might enhance the transmission and pathogenicity in wild birds [43]. However, there were limitations in this study, as many HPAIV cases in wild birds were reported through the passive survey for H5 HPAIV in Japan from the suspected dead birds. This may not represent the true ecology of the geographical distribution of three H5 HPAIV subgroups (G2b, G2c, and G2d) in Japan.

The unusually high number of dead crows positive for H5 HPAIVs in Hokkaido was also reported in winter 2022–2023, similar to winter 2021–2022 [15]. Interestingly, multiple H5 HPAIV genotypes, genotypes I, III, and IV (Figure 2), were identified among dead crows discovered in the city center of Hokkaido, in winter 2022–2023, indicating that crows could play a role in virus transmission by introducing various genotypes of HPAIVs from the outside to inside of the poultry farm. Many HPAI cases in crows may have led to virus spillover to foxes, as HPAIVs isolated from crows and foxes were genetically highly homologous to one another. The increase in spillover of HPAIVs from birds to mammals has also caused great concern for public health regarding the adaptation of HPAIVs in mammals.

The antigenicity of isolates in winter 2022–2023 and an isolate in November 2023 was close to those of clade 2.3.4.4b H5 HPAIVs isolated in Japan or the Democratic Republic of the Congo of Africa in the past few years and still close with H5 HPAIVs in clade 2.3.4.4c (Figure 3). In contrast, moderate differences in antigenicity between clades 2.3.4.4b and 2.3.4.4e H5 HPAIVs were observed, as supported by the previous study investigating clade 2.3.4.4b H5 HPAIVs in Japan and Africa [27, 30]. In addition, slight differences in antigenicity among isolates of three subgroups, G2b, G2c, and G2d, were observed, corresponding to an observation in a study among subgroups G2b and G2d of 2.3.4.4b H5 HPAIV isolates in Japan in winter 2021–2022 [9]. Since autumn 2021, the Eurasian lineage of H5 HPAIVs in clade 2.3.4.4b has been spreading globally and eventually introduced into countries such as the South American region, which are rarely affected by H5 HPAIVs in this lineage. The constant widespread of H5 HPAIV might generate HPAIVs with different characteristics, including antigenicity. However, the antigenicity of the isolates in Hokkaido has demonstrated minor antigenicity changes in the past several seasons, indicating preservation of H5 HPAIVs in clade 2.3.4.4b under less selective pressure for major antigenicity changes.

A low morbidity rate but a sudden increase in lethality rates in broiler flocks has been described during an outbreak of clade 2.3.4.4 b H5N1 HPAIV in Italy in winter 2021–2022 [44]. Thus, to understand the pathogenicity of H5 HPAIVs in different chicken breeds, four representative HPAIVs with different gene constellations were selected for pathogenicity study in three chicken breeds. In this study, it was highlighted that Chunky (broiler) chickens also demonstrated similar mortality rates and clinical signs to the ones found in White Leghorn (layer) and Rhode Island Red (layer) chickens except for one strain. The pathogenicity of Ck/Hok/B102/23 (H5N1) in Chunky chicken was significantly lower than in White Leghorn chicken, corresponding to the observation reported in Italy broiler flocks even though the virus load recovered from the swab samples among Chunky and White Leghorn chickens were similar (Tables 2 and 3). The difference in the pathogenicity displayed in different chicken breeds can be due to the existence of some avian genes such as PLAU, VCAM1, TNFRSF1A, and PGF gene, which were downregulated during inflammatory response in the lungs of the chicken breed, which can affect disease tolerance; the commercial broiler in Spain was reported to be resistant toward the clade 2.3.4.4b H5N8 HPAIV infection [45]. In this study, it is estimated that 50% of chicken lethal dose of Ck/Hok/B102/23 (H5N1) in Chunky chicken should be 10^5.7^ EID_50_, which is higher than the ones previously reported in White Leghorn chicken [9]. Furthermore, in the pathogenicity assessment of representative HPAIVs in all three chicken breeds, the pathogenicity of Ck/Hok/E001/22 (H5N1) was generally highest, followed by Cr/Hok/B003/22 (H5N2), lastly Ew/Hok/Q71/22 (H5N1) and Ck/Hok/B102/22 (H5N1). A significant difference was observed in the mean death time between Ck/Hok/E001/22 (H5N1) and Ew/Hok/Q71/22 (H5N1), corresponding to a slower virus growth kinetics in swab samples of Ew/Hok/Q71/22 (H5N1) (Tables 2 and 3 and Table S4). The slower growth kinetics of Ew/Hok/Q71/22 (H5N1) indicated the inability of the virus to replicate well in respiratory and intestinal organs compared to Ck/Hok/E001/22 (H5N1) and Cr/Hok/B003/22 (H5N2). This demonstrated that different genotypes of HPAIVs can contribute to different characteristics in pathogenicity even within the same clade 2.3.4.4b H5 HPAIVs, as lower pathogenicity was observed in chickens challenged with H5N8 HPAIV compared to H5N1 HPAIV that was circulating at the same time [9]. As different pathogenicity characteristics were observed among the representative viruses, the difference in the amino acid in each of the representative HPAIV strains was compared according to pathogenicity markers in previously reported AIVs [33]. All identified polymorphic sites in isolates have been linked to several associated phenotypes, such as altering the activities of the polymerase, replication efficacy in mammalian cells, and virulence in mammalian or avian hosts [33]. Interestingly, the relatively lower pathogenicity observed in chickens infected with Ew/Hok/Q71/22 (H5N1) might be due to the preservation of the amino acid (105M) in NP, as all isolates in this season, except for Ew/Hok/Q71/22 (H5N1) and Ew/Hok/M184/22 (H5N1) with the mutation (M105V) in NP, which could contribute to the increase in the pathogenicity of chickens [33, 42]. Though molecular determinants in contributing to different pathogenicity observed in four HPAIVs were unknown yet, it was speculated that viral segments originating from LPAIVs might play a role in contributing to switch virus pathogenicity, as some studies have demonstrated that reassortant viruses with PB2, PB1, PA, and NP genes could contribute to higher pathogenicity in ferrets [46]. As strain-specific mutations were observed across the substitution of polymerase units of the four representative viruses, the role of those unique amino acid residues observed across the investigated viruses might need to be clarified in future studies. Nevertheless, as a potential public health concern, the receptor binding sites of viral HA of all 30 isolates still indicate the preference to bind to *α*2,3-sialic acid linkage (237G, 238Q, and 240G; H5 numbering). Any associated markers contributing to high virulence in mammals were not found in Hokkaido isolates, demonstrating that threat of these viruses on public health should be very low [33]. However, cases among mink-to-mink transmission in Spain should heighten the concern about HPAIV adaptation in mammals if viruses continue circulating among wild birds and mammals globally [47].

Overall, H5 HPAIVs isolated in Hokkaido in winter 2022–2023 were phylogenetically, antigenically, and pathogenetically characterized. These were genetically close to Eurasian viruses isolated in winter 2021–2022 and consisted of multiple genotypes identified across wild birds and poultry, indicating the possibility of introducing HPAIVs with different genotypes by migratory birds to Japan. Furthermore, the same genotype of H5 HPAIV was also reintroduced to Hokkaido in early winter 2023–2024. Thus, the invasion of multiple viruses with distinct characteristics, especially transmissibility, host range, and pathogenicity in multiple animals, may perplex the understanding of the epidemiology and ecology of pathogens in the field to reduce their risk. Therefore, continuous monitoring and information sharing of the latest HPAIVs must be essential at the global level to understand dominant or critical HPAIV strains to establish effective control actions.

## Figures and Tables

**Table 1:** List of H5 high pathogenicity avian influenza viruses isolated in Hokkaido and eastern part of Russia in winter 2022–2023 and early winter 2023–2024.

ID	Virus	Subgroup	Genotype	Sample collection date	City/town	Latitude	Longitude	^1^Accession number of strain
1	A/Eurasian wigeon/Hokkaido/Q71/2022 (H5N1)	G2b	V	11/11/2022	Betsukai	43°36′12″N	145°17′35″E	EPI_ISL_15576617
2	A/Eurasian wigeon/Hokkaido/M184/2022 (H5N1)	G2b	V	27/11/2022	Monbetsu	44°25′47″N	143°50′25″E	EPI_ISL_15732766
3	A/large-billed crow/Hokkaido/B003/2022 (H5N2)	G2d	III	28/10/2022	Sapporo	43°03′50″N	141°20′35″E	EPI_ISL_15732741
4	A/slaty-backed gull/Hokkaido/011M111/2022 (H5N1)	G2d	I	06/11/2022	Shari	43°56′17″N	144°42′43″E	EPI_ISL_16698579
5	A/slaty-backed gull/Hokkaido/011M114/2022 (H5N1)	G2d	I	16/11/2022	Abashiri	44°01′15″N	144°16′24″E	EPI_ISL_16955798
6	A/red-crowned crane/Hokkaido/20221120001/2022 (H5N1)	G2d	I	20/11/2022	Tobetsu	43°13′47″N	141°31′32″E	EPI_ISL_16698576
7	A/white-tailed eagle/Hokkaido/20221123001/2022 (H5N1)	G2d	I	23/11/2022	Urahoro	42°48′33″N	143°39′30″E	EPI_ISL_16955829
8	A/large-billed crow/Hokkaido/011E084/2022 (H5N1)	G2d	I	24/11/2022	Mukawa	42°37′31″N	142°00′32″E	EPI_ISL_16955748
9	A/large-billed crow/Hokkaido/0112P083−2/2022 (H5N1)	G2d	I	05/12/2022	Kushiro	42°57′14″N	144°31′56″E	EPI_ISL_16955765
10	A/large-billed crow/Hokkaido/B004/2022 (H5N1)	G2d	IV	12/12/2022	Sapporo	43°03′50″N	141°20′35″E	EPI_ISL_17267427
11	A/large-billed crow/Hokkaido/B00B/2022 (H5N1)	G2d	IV	18/12/2022	Sapporo	43°03′50″N	141°20′35″E	EPI_ISL_17317372
12	A/large-billed crow/Hokkaido/B00C/2022 (H5N1)	G2d	IV	19/12/2022	Sapporo	43°03′50″N	141°20′35″E	EPI_ISL_17317373
13	A/large-billed crow/Hokkaido/B005/2022 (H5N1)	G2d	I	21/12/2022	Sapporo	43°04′50″N	141°20′26″E	EPI_ISL_17267428
14	A/large-billed crow/Hokkaido/B006/2023 (H5N1)	G2d	IV	04/02/2023	Sapporo	43°03′50″N	141°20′35″E	EPI_ISL_17316075
15	A/large-billed crow/Hokkaido/B007/2023 (H5N1)	G2d	IV	13/03/2023	Sapporo	43°03′50″N	141°20′35″E	EPI_ISL_17316489
16	A/large-billed crow/Hokkaido/B008/2023 (H5N1)	G2d	IV	13/03/2023	Sapporo	43°03′50″N	141°20′35″E	EPI_ISL_17316965
17	A/large-billed crow/Hokkaido/0103E088/2023 (H5N1)	G2d	I	24/03/2023	Mukawa	42°37′31″N	142°00′32″E	EPI_ISL_17950087
18	A/large-billed crow/Hokkaido/0103E089/2023 (H5N1)	G2d	I	29/03/2023	Mukawa	42°37′31″N	142°00′32″E	EPI_ISL_17950253
19	A/large-billed crow/Hokkaido/B049/2023 (H5N1)	G2d	IV	11/04/2023	Sapporo	43°03′50″N	141°20′35″E	EPI_ISL_17950254
20	A/large-billed crow/Hokkaido/B052/2023 (H5N1)	G2d	I	13/04/2023	Sapporo	43°03′50″N	141°20′35″E	EPI_ISL_18007229
21	A/large-billed crow/Hokkaido/B053/2023 (H5N1)	G2d	IV	18/04/2023	Sapporo	43°03′50″N	141°20′35″E	EPI_ISL_18007230
22	A/large-billed crow/Hokkaido/B067/2023 (H5N1)	G2d	I	23/11/2023	Sapporo	43°03′50″N	141°20′35″E	EPI_ISL_18591747
23	A/chicken/Hokkaido/HU-E001/2022 (H5N1)	G2d	II	07/11/2022	Atsuma	42°43′26″N	141°52′42″E	EPI_ISL_16698060
24	A/chicken/Hokkaido/HU-E008/2022 (H5N1)	G2d	I	11/11/2022	Date	42°30′44″N	140°52′03″E	EPI_ISL_16698477
25	A/chicken/Hokkaido/HU-E010/2022 (H5N1)	G2d	I	11/11/2022	Date	42°30′44″N	140°52′03″E	EPI_ISL_17267420
26	A/chicken/Hokkaido/HU-B102/2023 (H5N1)	G2c	VI	27/03/2023	Chitose	42°49′15″N	141°39′05″E	EPI_ISL_17638141
27	A/chicken/Hokkaido/HU-B202/2023 (H5N1)	G2c	VI	02/04/2023	Chitose	42°49′15″N	141°39′05″E	EPI_ISL_17638143
28	A/chicken/Hokkaido/HU-B301/2023 (H5N1)	G2c	VI	06/04/2023	Chitose	42°49′15″N	141°39′05″E	EPI_ISL_17638448
29	A/Ezo red fox/Hokkaido/3/2023 (H5N1)	G2d	IV	20/02/2023	Sapporo	43°03′50″N	141°20′35″E	EPI_ISL_17857959
30	A/Ezo red fox/Hokkaido/2/2023 (H5N1)	G2d	IV	12/04/2023	Sapporo	43°03′50″N	141°20′35″E	EPI_ISL_17766057
31	A/crow/Khabarovsk/776-56/2022 (H5N1)	G2d	I	15/04/2022	Khabarovsk	50°39′12″N	136°57′16″E	EPI_ISL_18074199
32	A/goose/Magadan/2272-5/2022 (H5N1)	G2d	I	10/09/2022	Magadan	59°32′41″N	150°53′08″E	EPI_ISL_18071580

^1^The Global Initiative on Sharing All Influenza Data accession numbers.

**Table 2:** Virus recovery from swabs collected in Chunky chickens after postinoculation with four representative viruses.

Viruses	Days on dead	Virus titers in swab sample (log_10_ TCID_50_/mL)													
		Day 1		Day 2		Day 3		Day 4		Day 5		Day 6		Day 7	
		Tracheal	Cloacal	Tracheal	Cloacal	Tracheal	Cloacal	Tracheal	Cloacal	Tracheal	Cloacal	Tracheal	Cloacal	Tracheal	Cloacal
Ck/Hok/E001/22 (H5N1)	2	1.4	2.7	5.5	3.7	—^1^	—	—	—	—	—	—	—	—	—
	2	2.5	3.5	4.7	5.0	—	—	—	—	—	—	—	—	—	—
	2	<0.5^2^	<0.5	5.0	3.7	—	—	—	—	—	—	—	—	—	—
	2	2.0	2.2	4.7	4.3	—	—	—	—	—	—	—	—	—	—

Cr/Hok/B003/22 (H5N2)	2	<0.5	<0.5	5.5	4.7	—	—	—	—	—	—	—	—	—	—
	2	<0.5	<0.5	6.0	5.0	—	—	—	—	—	—	—	—	—	—
	2	<0.5	<0.5	5.5	3.5	—	—	—	—	—	—	—	—	—	—
	3	<0.5	<0.5	1.5	<0.5	5.7	3.5	—	—	—	—	—	—	—	—

Ew/Hok/Q71/22 (H5N1)	5	<0.5	<0.5	<0.5	<0.5	2.5	<0.5	3.7	2.3	2.7	1.7	—	—	—	—
	3	<0.5	<0.5	3.5	1.5	3.3	3.5	—	—	—	—	—	—	—	—
	3	<0.5	<0.5	3.5	2.0	3.5	2.5	—	—	—	—	—	—	—	—
	3	<0.5	<0.5	4.3	2.0	4.0	2.0	—	—	—	—	—	—	—	—

Ck/Hok/B102/23 (H5N1)	4	<0.5	<0.5	1.8	<0.5	3.0	2.7	4.5	2.3	—	—	—	—	—	—
	4	<0.5	<0.5	<0.5	<0.5	<0.5	<0.5	4.5	4.7	—	—	—	—	—	—
	3	<0.5	<0.5	4.3	4.3	4.3	4.0	—	—	—	—	—	—	—	—
	14	<0.5	<0.5	<0.5	<0.5	<0.5	<0.5	<0.5	<0.5	<0.5	<0.5	<0.5	<0.5	<0.5	<0.5

^1^Indicate no samples were collected. ^2^<0.5 log_10_ TCID_50_/mL is the lowest detection limit of the virus titers.

**Table 3:** Virus recovery from swabs collected in White Leghorn chickens after postinoculation with four representative viruses.

Viruses	Days on dead	Virus titers in swab sample (log_10_ TCID_50_/mL)									
		Day 1		Day 2		Day 3		Day 4		Day 5	
		Tracheal	Cloacal	Tracheal	Cloacal	Tracheal	Cloacal	Tracheal	Cloacal	Tracheal	Cloacal
Ck/Hok/E001/22 (H5N1)	2	1.5	2.0	3.7	2.3	—^1^	—	—	—	—	—
	2	2.0	2.7	3.7	1.7	—	—	—	—	—	—
	2	4.0	1.3	4.7	3.0	—	—	—	—	—	—
	2	2.5	<0.5^2^	4.3	3.7	—	—	—	—	—	—

Cr/Hok/B003/22 (H5N2)	2	2.5	<0.5	6.0	3.5	—	—	—	—	—	—
	2	<0.5	<0.5	4.0	3.0	—	—	—	—	—	—
	2	2.3	2.3	4.0	3.5	—	—	—	—	—	—
	4	<0.5	<0.5	1.7	<0.5	3.7	2.7	—	—	—	—

Ew/Hok/Q71/22 (H5N1)	5	<0.5	<0.5	<0.5	<0.5	1.0	<0.5	2.7	<0.5	2.7	1.5
	5	<0.5	<0.5	1.0	<0.5	1.7	<0.5	2.0	<0.5	2.7	1.8
	5	<0.5	<0.5	<0.5	<0.5	<0.5	<0.5	3.5	1.7	2.7	2.0
	5	<0.5	<0.5	<0.5	1.0	1.3	1.5	3.5	2.0	3.5	1.7

Ck/Hok/B102/23 (H5N1)	2	<0.5	<0.5	3.7	3.0	—	—	—	—	—	—
	2	<0.5	<0.5	4.0	2.7	—	—	—	—	—	—
	2	<0.5	<0.5	4.5	2.7	—	—	—	—	—	—
	4	<0.5	<0.5	<0.5	<0.5	2.7	2.7	2.3	2.3	—	—

^1^Indicate no samples were collected. ^2^<0.5 log_10_ TCID_50_/mL is the lowest detection limit of the virus titers.

**Table 4:** Mutational analysis of H5 high pathogenicity avian influenza viruses isolated in Hokkaido in winter 2022–2023 and early winter 2023–2024.

Segment	Mutation	Amino acid in the following number of virus^1^																														Associated phenotypes	References
		1	2	3	4	5	6	7	8	9	10	11	12	13	14	15	16	17	18	19	20	21	22	23	24	25	26	27	28	29	30		
PB2	I292V	I	•^2^	•	•	V	V	V	•	V	V	V	•	•	V	•	V	•	•	V	•	V	•	•	•	•	•	•	•	V	V	Enhanced polymerase activity, increased virulence in mice	[37]

PB1	K207R	K	•	R	•	•	R	R	R	•	•	•	•	•	•	•	•	•	•	•	•	•	•	•	R	R	•	•	•	•	•	Decreased polymerase activity in mammalian cell line	[41]

PB1-F2	N66S	N	•	S	S	S	S	S	S	S	S	S	S	S	S	S	S	S	S	S	S	S	S	S	S	S	S	S	S	S	S	Enhanced polymerase activity, virulence and antiviral response in mice	[36]

PA	S224P	S	•	•	P	P	•	•	P	•	•	•	•	•	•	•	•	•	•	•	P	•	•	•	•	•	•	•	•	•	•	Enhanced polymerase activity and virulence in mice and duck	[38]
	Q400P	Q	•	•	P	P	P	P	P	P	•	•	•	P	•	•	•	P	P	•	P	•	P	P	P	P	P	P	P	•	•	Decreased virulence in mice	[38]

NP	M105V	M	•	V	V	V	V	V	V	V	V	V	V	V	V	V	V	V	V	V	V	V	V	V	V	V	V	V	V	V	V	Increased virulence in chickens	[42]
	N319K	N	•	K	K	K	K	K	K	K	K	K	K	K	K	K	K	K	K	K	K	K	K	K	K	K	•	•	•	K	K	Increased polymerase activity and replication in mammalian	[39]

^1^The number of the virus is corresponding to ID in Table 1. ^2^Dot indicates same amino acid in virus number 1.

## Data Availability

The data presented in this study are openly shared in the Global Initiative on Sharing All Influenza Data. All the accession numbers of the virus sequence data are described in Table 1.

## Supplementary Materials

Supplementary Materials Table S1: primer sets for avian influenza virus genome amplification of polymerase gene segments. Table S2: BLAST search results of H5 high pathogenicity avian influenza viruses in Hokkaido in 2022–2023 in the GISAID database. Table S3: cross-hemagglutination inhibition assay of H5 high pathogenicity avian influenza viruses in Hokkaido in winter 2022–2023 and early winter 2023–2024. Table S4: virus recovery from swabs collected in Rhode Island Red chickens after post-inoculation with three representative viruses. Table S5: mutational analysis on all the reported amino acid positions of H5 high pathogenicity avian influenza viruses isolated in Hokkaido in winter 2022–2023 and early winter 2023–2024. Figure S1: phylogenetic tree analysis of internal genes of H5 high pathogenicity avian influenza viruses isolated in the Far East in winter 2022–2023 and early winter 2023–2024.
