# Supplementary material for: Continuous Introduction of H5 High Pathogenicity Avian Influenza Viruses in Hokkaido, Japan: Characterization of Viruses Isolated in Winter 2022–2023 and Early Winter 2023–2024
Source: Transbound Emerg Dis. 2024 Mar 14;2024:1199876. doi: 10.1155/2024/1199876 (PMC12016768; doi:10.1155/2024/1199876)

## List of Tables and Figures

Supplemental Table 1: Primer sets for avian influenza virus genome amplification of polymerase gene segments.

Supplemental Table 2: BLAST search results of H5 high pathogenicity avian influenza viruses in Hokkaido in 2022–2023 in the GISAID database.

Supplemental Table 3: Cross-hemagglutination inhibition assay of H5 high pathogenicity avian influenza viruses in Hokkaido in winter 2022–2023 and early winter 2023–2024.

Supplemental Table 4: Virus recovery from swabs collected in Rhode Island Red chickens after post-inoculation with three representative viruses.

Supplemental Table 5: Mutational analysis on all the reported amino acid positions of H5 high pathogenicity avian influenza viruses isolated in Hokkaido in winter 2022–2023 and early winter 2023–2024.

Supplemental Figure 1: Phylogenetic tree analysis of internal genes of H5 high pathogenicity avian influenza viruses isolated in the Far East in winter 2022–2023 and early winter 2023–2024. Phylogenetic tree analysis on the internal gene segments based on (A) polymerase basic protein 2, (B) polymerase basic protein 1, (C) polymerase acidic, (D) nucleoprotein, (E) matrix protein, and (F) non-structural protein segments of 29 H5 HPAIVs and an H5 HPAIV isolated in Hokkaido in winter 2022–2023 and early winter 2023–2024, respectively, together with the reference strains in clade 2.3.4.4b and subclades were analyzed using the maximum-likelihood method with MEGA 7. The scale bar represents the number of nucleotide substitutions per site. The number of nodes indicates the probability of confidence levels according to bootstrap analysis based on 1000 replicates >60%. H5 HPAIVs isolated in Hokkaido in 2022–2023 and early winter 2023–2024 are shown in red, H5 HPAIVs isolated in eastern Russia in 2022 are in green, and H5 HPAIVs isolated in Honshu Island in 2022–2023 are in blue. The HPAIVs isolated in this study were marked with a circle, and an isolate in early winter 2023–2024 was marked with double circles.


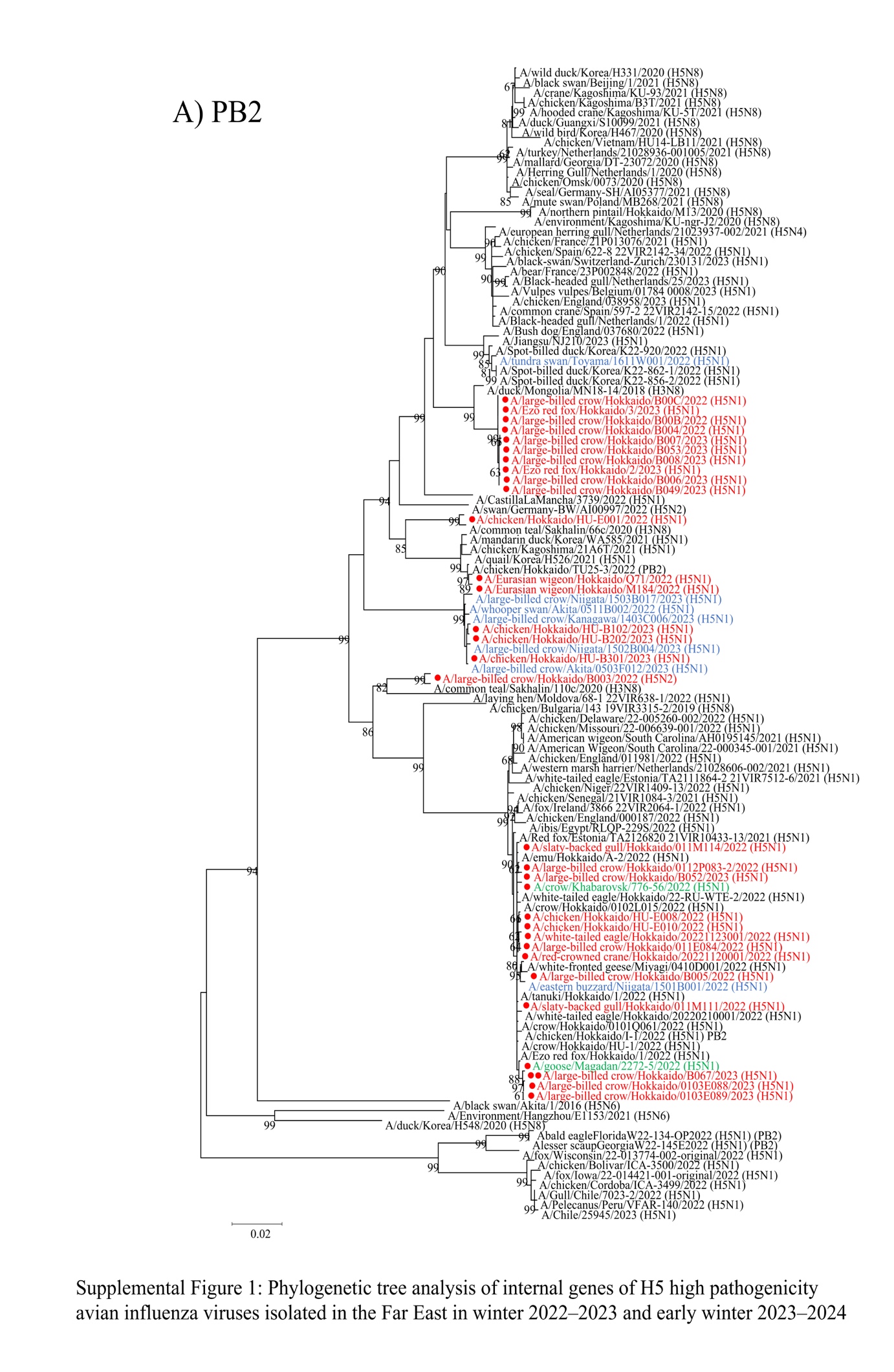


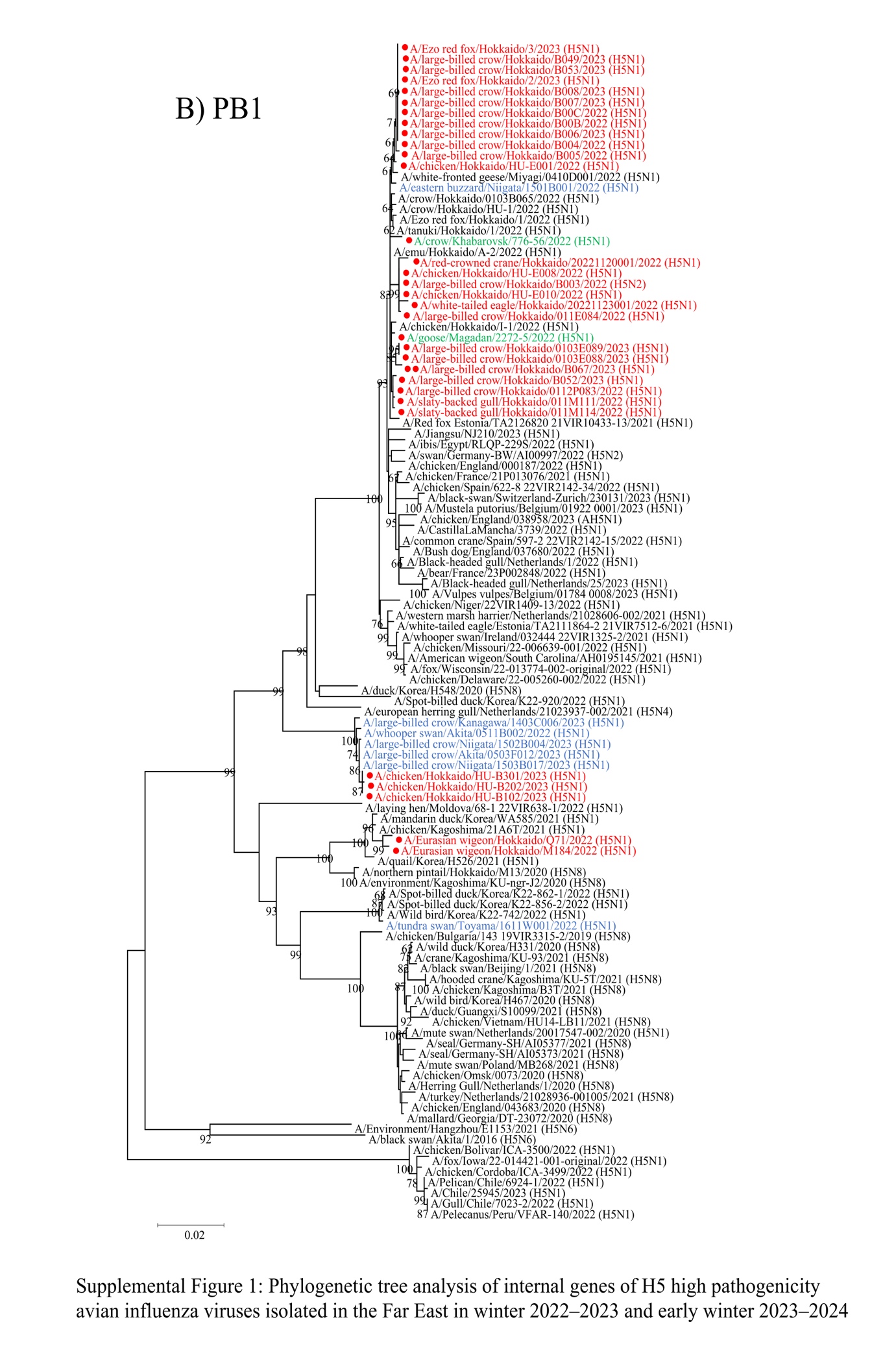


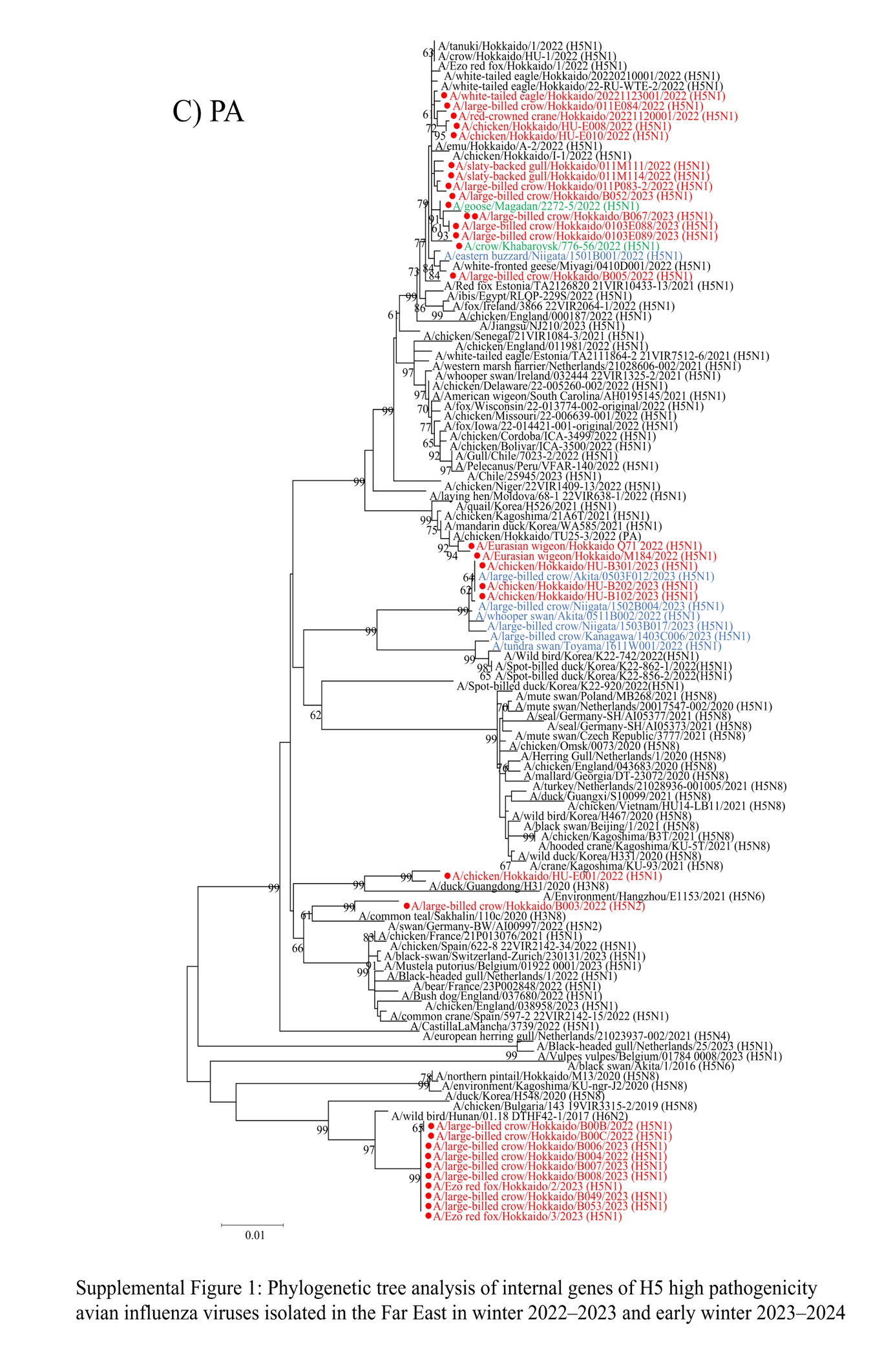


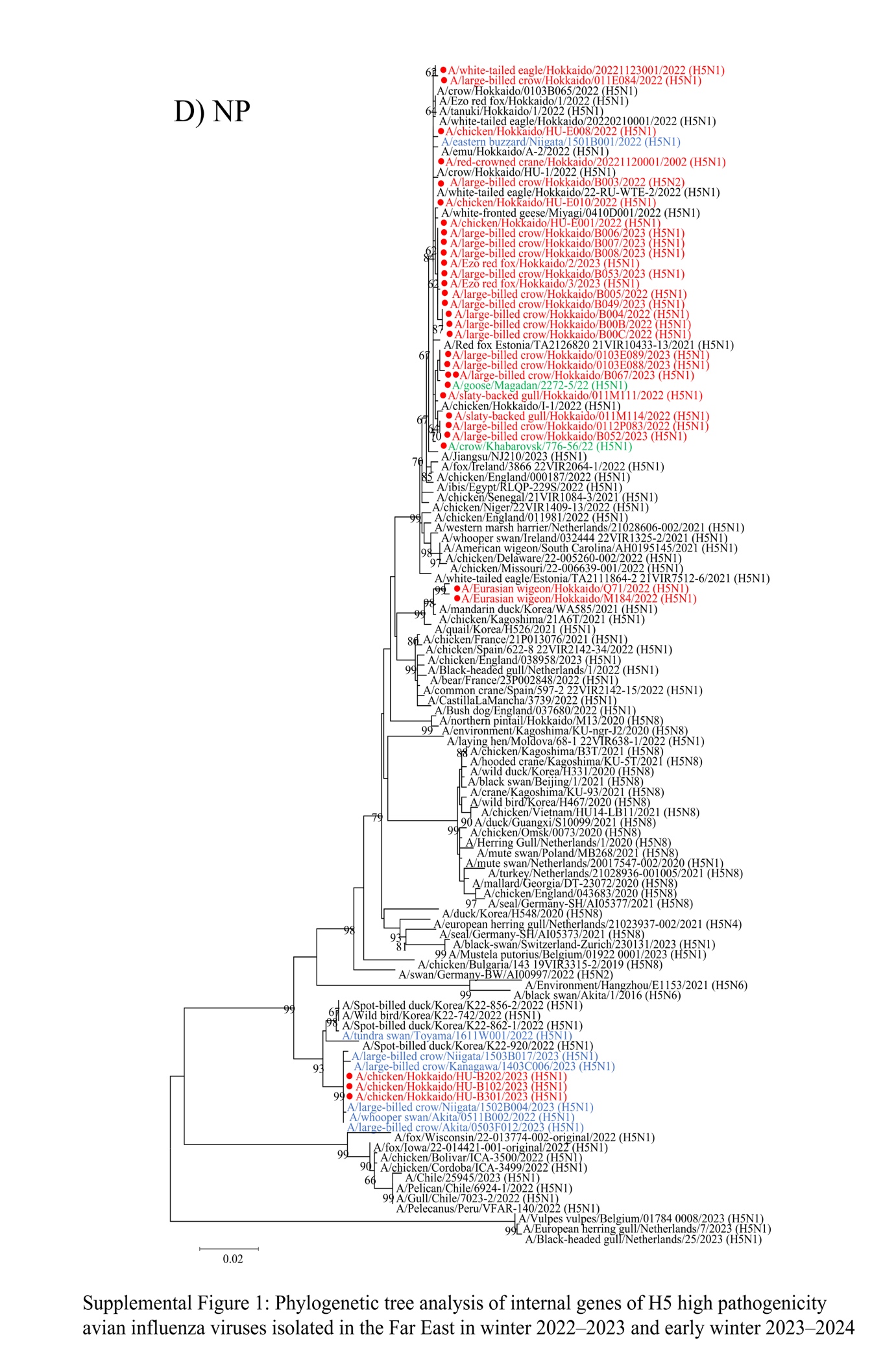


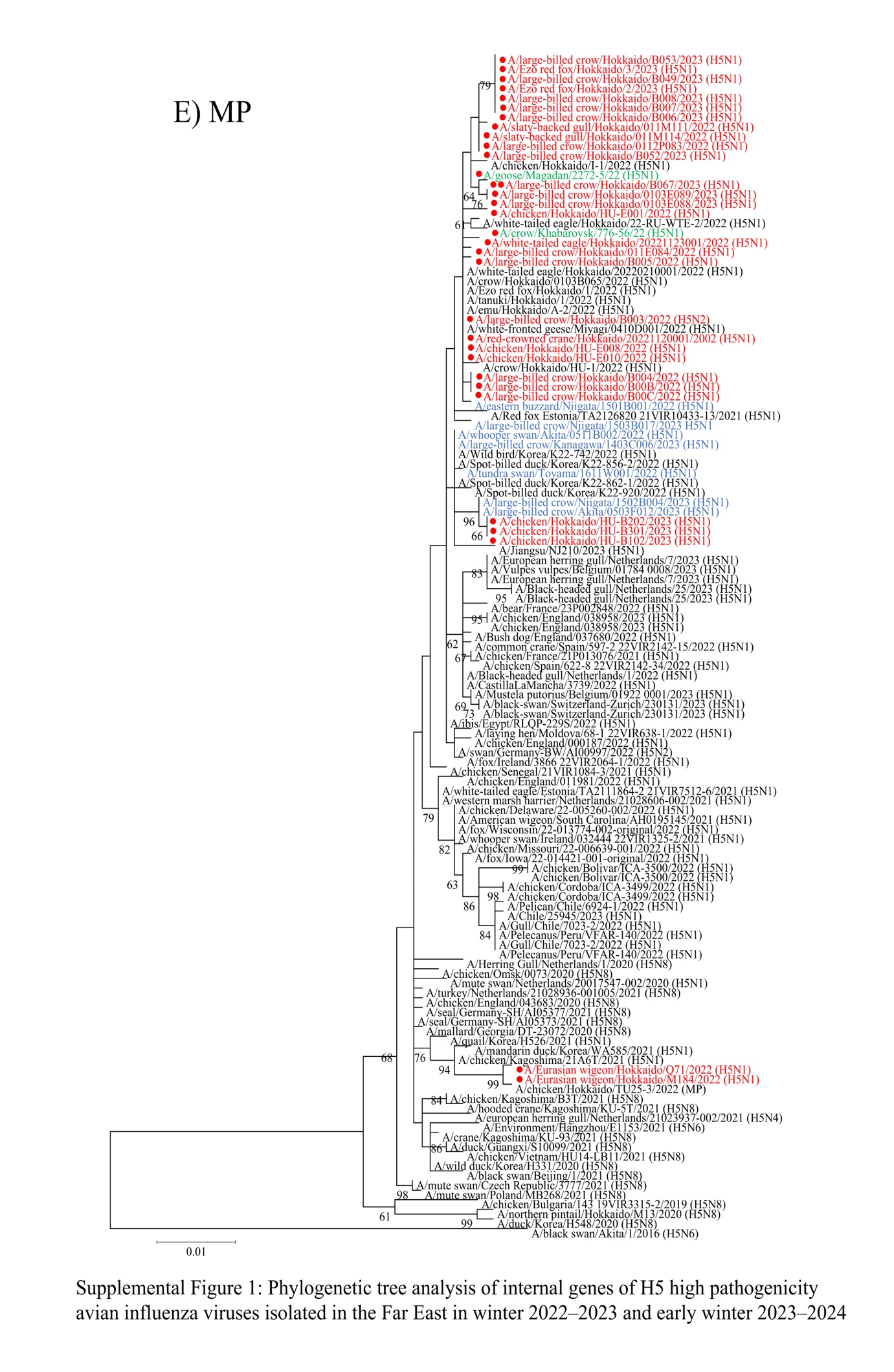


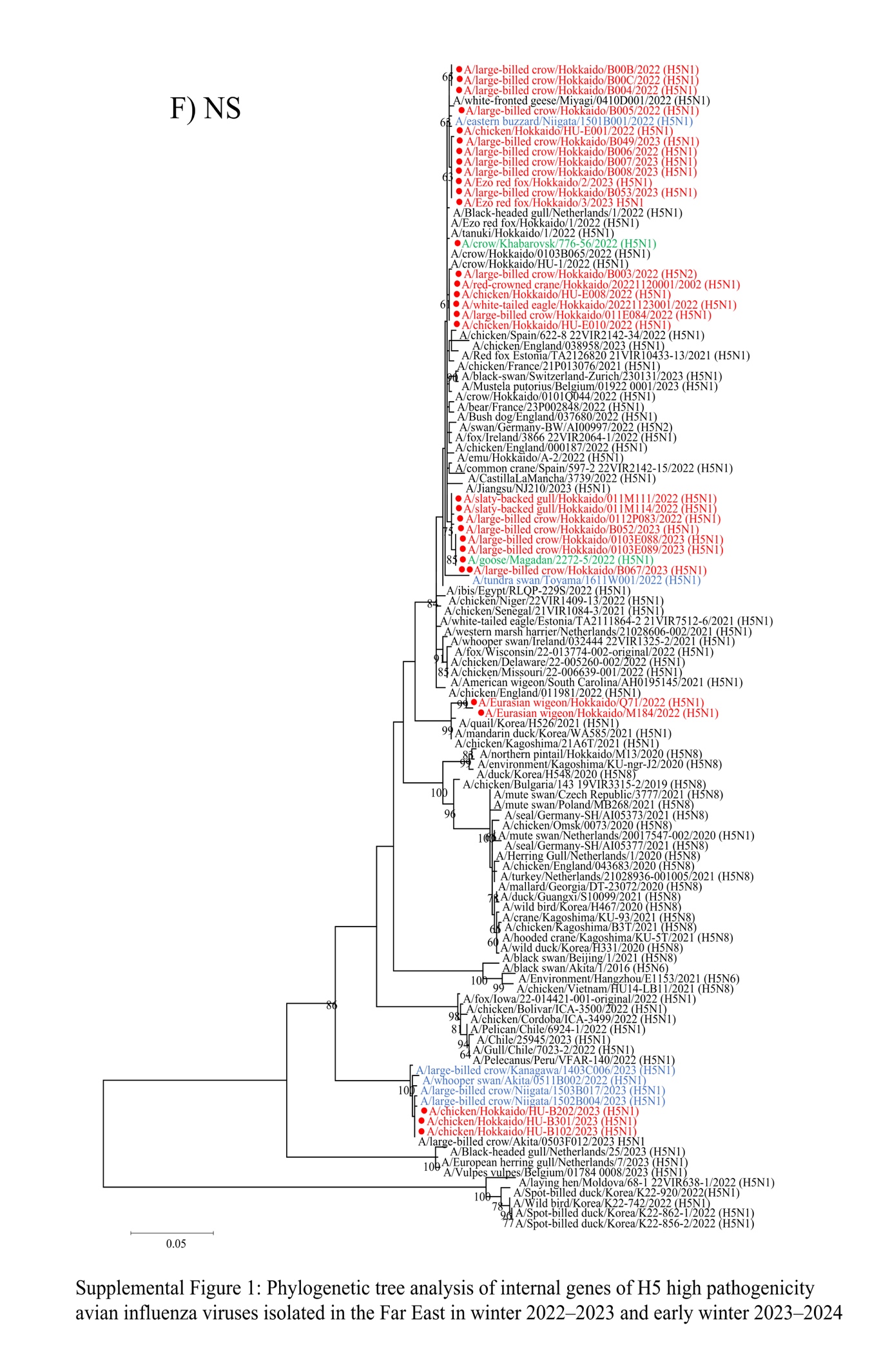


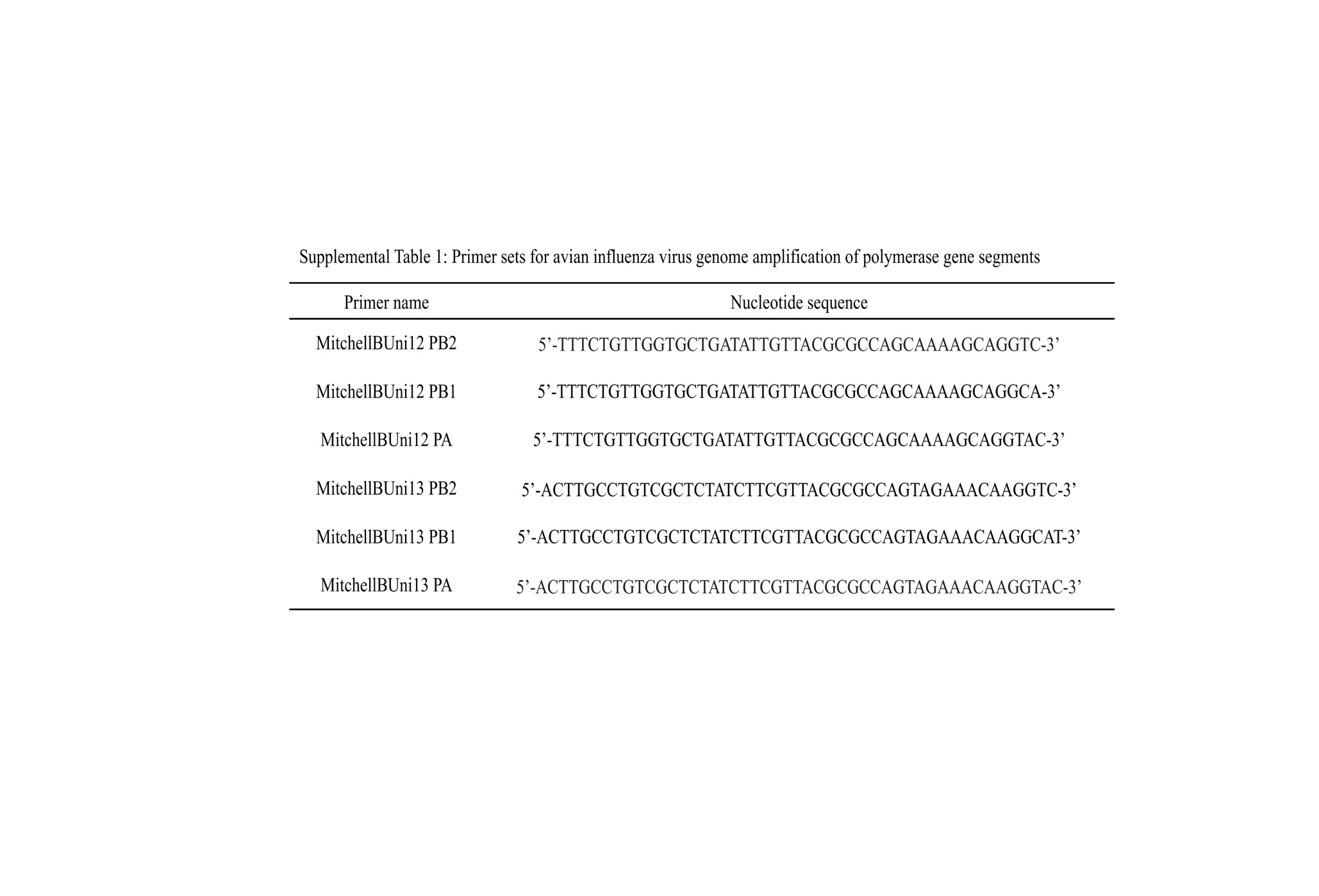


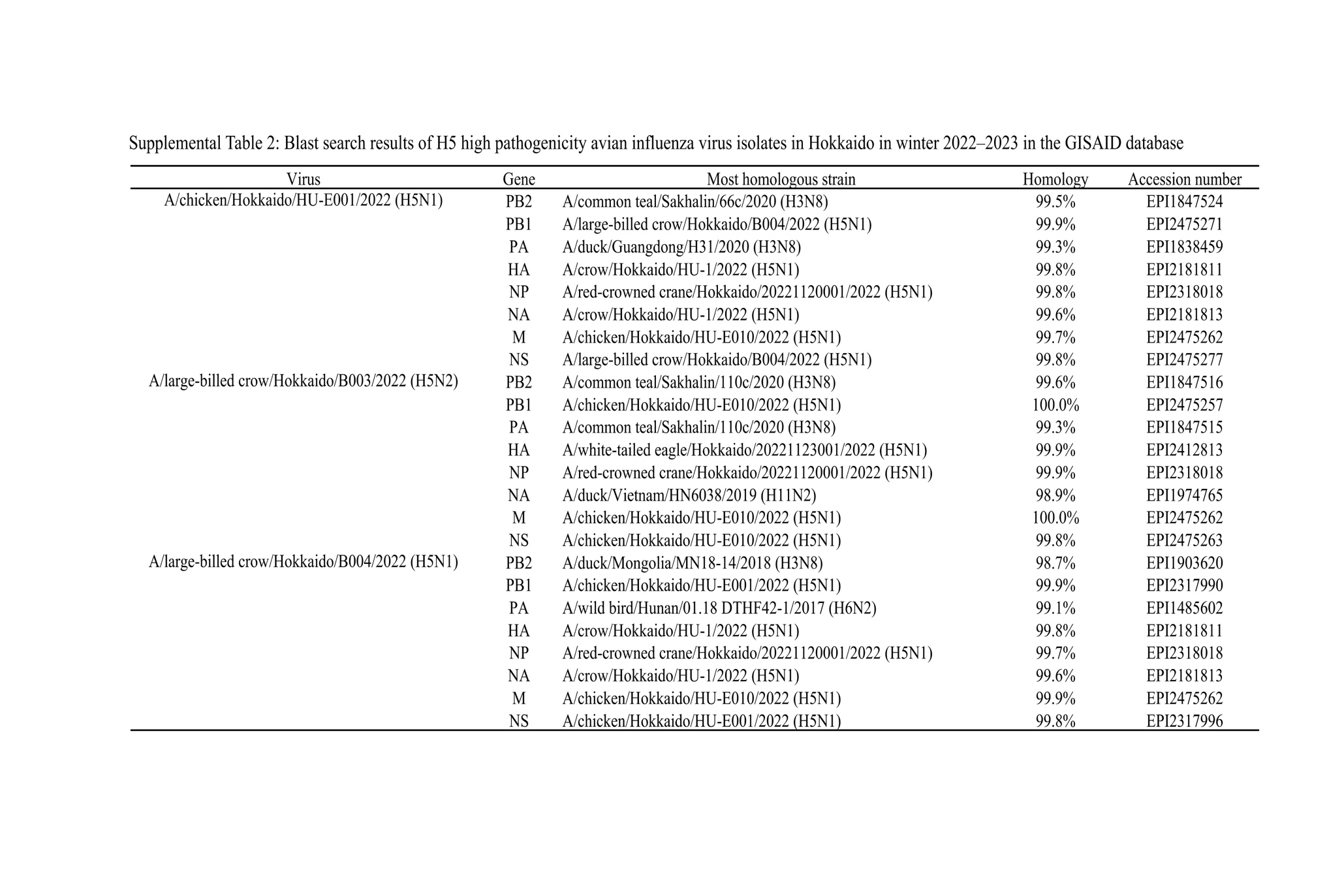


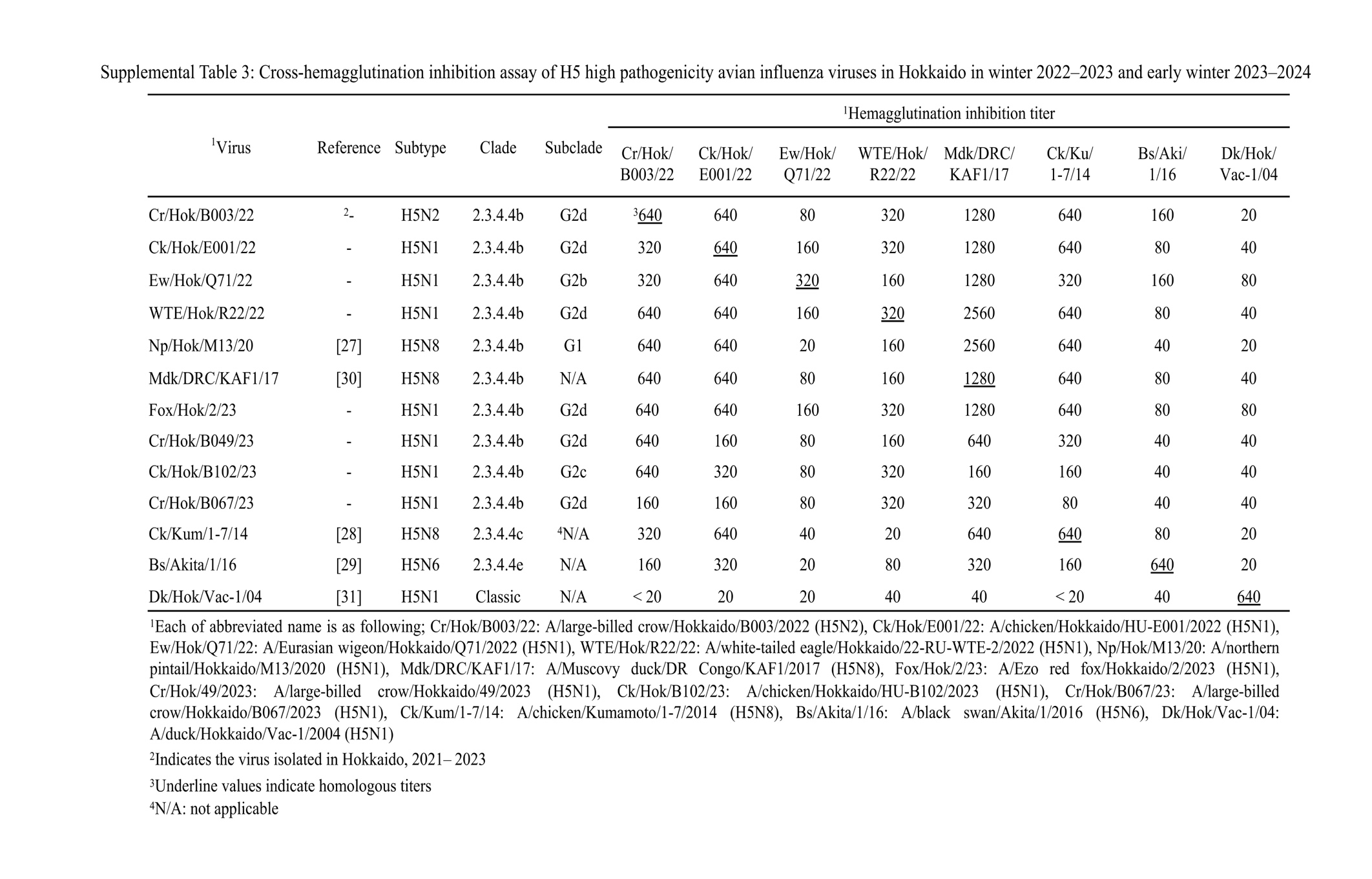


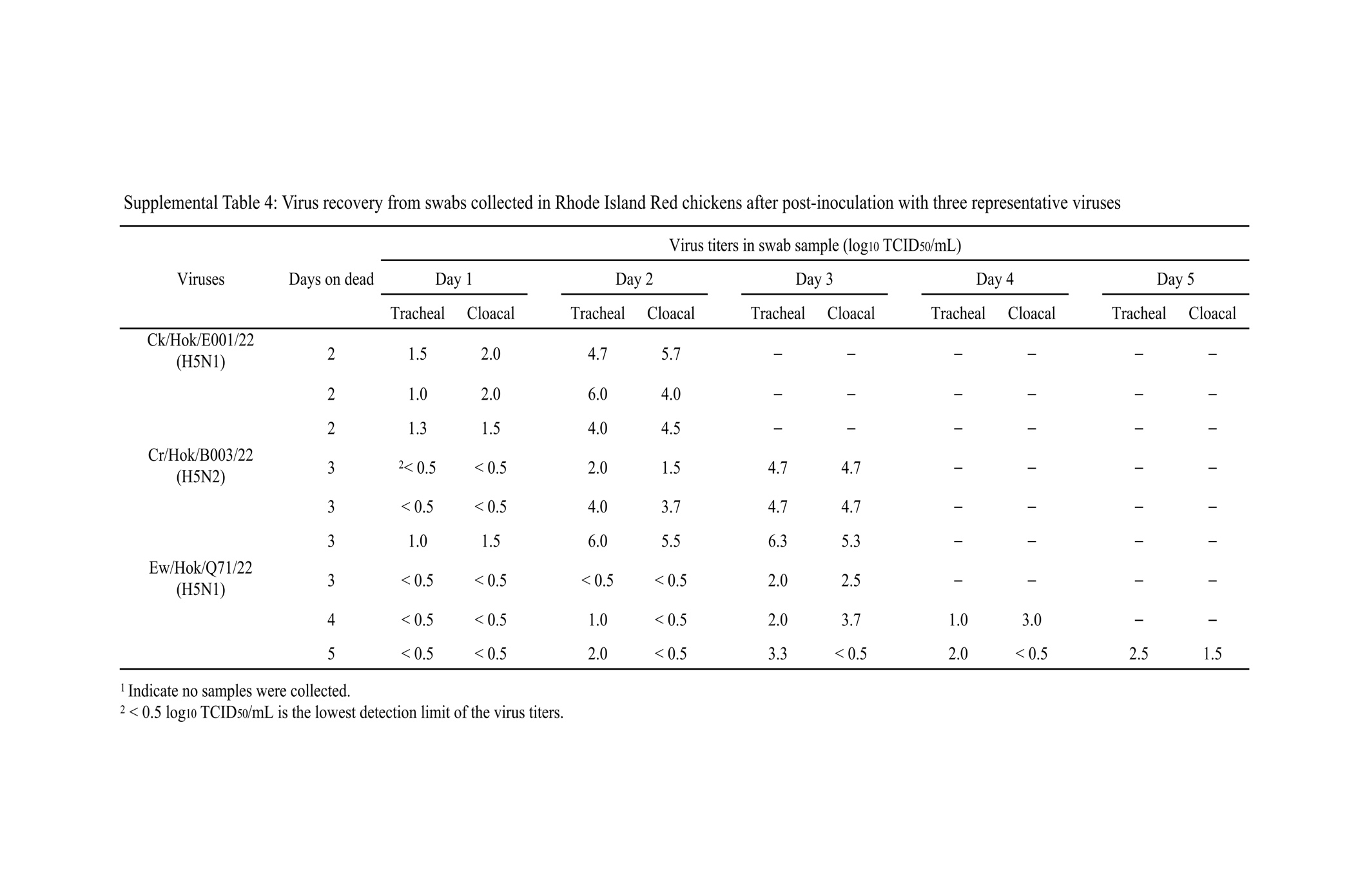


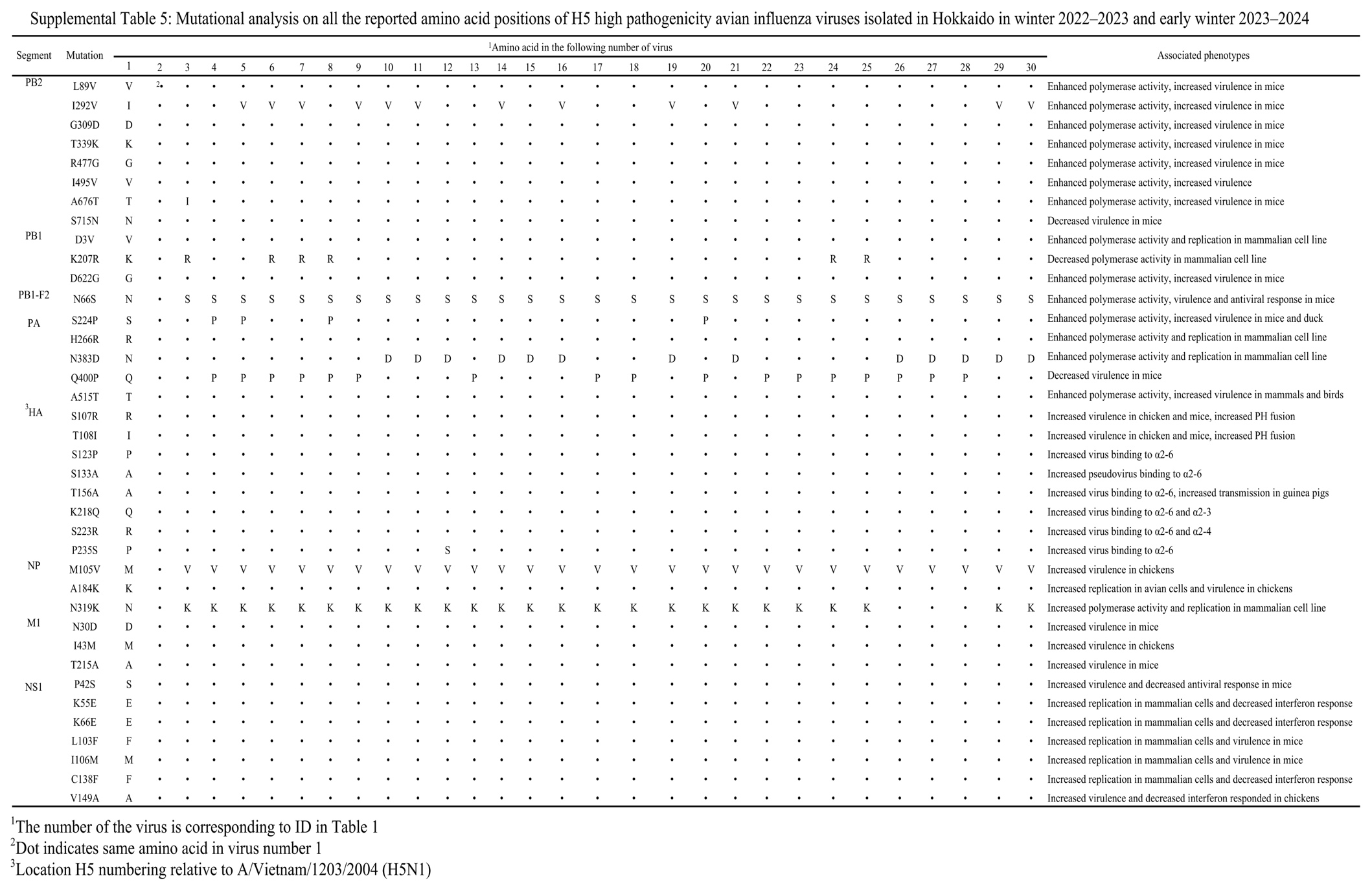

Supplement: Supplementary Materials — Table S1: primer sets for avian influenza virus genome amplification of polymerase gene segments. Table S2: BLAST search results of H5 high pathogenicity avian influenza viruses in Hokkaido in 2022–2023 in the GISAID database. Table S3: cross-hemagglutination inhibition assay of H5 high pathogenicity avian influenza viruses in Hokkaido in winter 2022–2023 and early winter 2023–2024. Table S4: virus recovery from swabs collected in Rhode Island Red chickens after post-inoculation with three representative viruses. Table S5: mutational analysis on all the reported amino acid positions of H5 high pathogenicity avian influenza viruses isolated in Hokkaido in winter 2022–2023 and early winter 2023–2024. Figure S1: phylogenetic tree analysis of internal genes of H5 high pathogenicity avian influenza viruses isolated in the Far East in winter 2022–2023 and early winter 2023–2024. [file 1199876.f1.docx]
